# The transport activity of the multidrug ABC transporter BmrA does not require a wide separation of the nucleotide-binding domains

**DOI:** 10.1016/j.jbc.2023.105546

**Published:** 2023-12-09

**Authors:** Margot Di Cesare, Elise Kaplan, Julia Rendon, Guillaume Gerbaud, Sepideh Valimehr, Alexia Gobet, Thu-Anh Thi Ngo, Vincent Chaptal, Pierre Falson, Marlène Martinho, Pierre Dorlet, Eric Hanssen, Jean-Michel Jault, Cédric Orelle

**Affiliations:** 1 Bacterial Nucleotide-Binding Proteins Team, Molecular Microbiology and Structural Biochemistry (MMSB), UMR 5086 CNRS/University of Lyon, Lyon, France; 2 CNRS, Aix-Marseille Université, BIP, IMM, Marseille, France; 3 Ian Holmes Imaging Center and Department of Biochemistry and Pharmacology and ARC Centre for Cryo-Electron Microscopy of Membrane Proteins, Bio21 Institute, University of Melbourne, Parkville, VIC, Australia; 4 Drug Resistance and Membrane Proteins Team, Molecular Microbiology and Structural Biochemistry (MMSB), UMR 5086 CNRS/University of Lyon, Lyon, France

**Keywords:** ABC transporter, multidrug resistance, antibiotics, conformational changes, catalytic cycle, transport cycle, drug transport, efflux pump

## Abstract

ATP-binding cassette (ABC) transporters are ubiquitous membrane proteins responsible for the translocation of a wide diversity of substrates across biological membranes. Some of them confer multidrug or antimicrobial resistance to cancer cells and pathogenic microorganisms, respectively. Despite a wealth of structural data gained in the last two decades, the molecular mechanism of these multidrug efflux pumps remains elusive, including the extent of separation between the two nucleotide-binding domains (NBDs) during the transport cycle. Based on recent outward-facing structures of BmrA, a homodimeric multidrug ABC transporter from *Bacillus subtilis*, we introduced a cysteine mutation near the C-terminal end of the NBDs to analyze the impact of disulfide-bond formation on BmrA function. Interestingly, the presence of the disulfide bond between the NBDs did not prevent the ATPase, nor did it affect the transport of Hoechst 33342 and doxorubicin. Yet, the 7-amino-actinomycin D was less efficiently transported, suggesting that a further opening of the transporter might improve its ability to translocate this larger compound. We solved by cryo-EM the apo structures of the cross-linked mutant and the WT protein. Both structures are highly similar, showing an intermediate opening between their NBDs while their C-terminal extremities remain in close proximity. Distance measurements obtained by electron paramagnetic resonance spectroscopy support the intermediate opening found in these 3D structures. Overall, our data suggest that the NBDs of BmrA function with a tweezers-like mechanism distinct from the related lipid A exporter MsbA.

ATP-binding cassette (ABC) transporters are ubiquitous membrane proteins that couple the energy of ATP binding and hydrolysis to the translocation of a wide variety of molecules across biological membranes. They can be categorized into three main classes: importers, exporters and mechanotransducers (1). Notwithstanding the incredible diversity of functions and structures of ABC transporters (2, 3, 4), they have been recently proposed to be classified into seven types according to their folding (1, 5). These classes are minimally made of two transmembrane domains (TMDs) and two nucleotide-binding domains (NBDs) that can be synthesized as a single polypeptide or up to four polypeptides. While the NBDs are well-conserved motor domains, the TMDs are highly divergent as they usually form the translocation pathway for the transported molecule(s). In humans, mutations of ABC proteins can cause a multitude of diseases and disorders (6, 7, 8). Furthermore, the overexpression of some ABC transporters can be responsible for multidrug resistance in cancer cells (9) and microorganisms such as bacteria (10), fungi (11, 12) or protozoan parasites (13, 14). Remarkable progress has been made in the last two decades toward the understanding of the molecular basis of ABC transporter function, especially with the increasing number of structures solved by single-particle cryo-EM (15). However, the mechanistic interpretation of the structures can be delicate and many questions remain unanswered regarding the molecular mechanism of transport, especially for multidrug transporters (16, 17). While they classically function according to an alternating access mechanism between inward-facing (IF) and outward-facing (OF) conformations, how the catalytic cycle is coupled with the translocation of such a wide diversity of substrates remains elusive. The extent of the nucleotide-binding domains (NBDs) separation in the IF state seems to be variable depending on the transporter and its translocated substrates (Fig. 1). This might also be influenced by the detergent or lipid environment (17, 18). The flexibility of multidrug transporters allows them to explore multiple states that may contribute to the accommodation of the substrates (19, 20, 21, 22). The opening and closing motions of the NBDs are indeed directly translated to movements in the transmembrane helices that contribute to the remodeling of the drug-binding pocket (23). Upon ATP binding, the NBDs engage in a transient tight dimer where the two nucleotides are sandwiched between the Walker A motif from one NBD and the ABC signature motif from the other NBD, thereby generating the OF conformation that is prone to drug release (24). The hydrolysis of ATP is then required to dissociate the tightly closed NBD dimer and to reset the transporter back to its IF conformation. In a large number of exporter structures solved in the absence of nucleotides, the NBDs do not contact each other and are separated by variable degrees (17). Although the widely open structure of the lipid A exporter MsbA was often considered nonphysiological, this state was recently found to be predominantly populated in *Escherichia coli* cells (18). Here, we investigated whether a large separation of the nucleotide-binding domains is required for the transport cycle of BmrA, a homodimeric multidrug ABC transporter from *Bacillus subtilis* (25). For this purpose, we introduced by site-directed mutagenesis a cysteine residue near the C-terminal end of the NBD to restrain the separation between the two NBDs during the catalytic cycle and analyzed the impact of the disulfide-bond formation on the ATPase and transport activities of BmrA. To understand the structural basis for the functional activity of the disulfide cross-linked mutant, we solved by cryo-EM its structure in the IF state. To evaluate if our observations hold true for the WT protein, we also resolved its IF structure. Both the WT and the cross-linked mutant showed similar 3D structures in the apo state and the opening between the two monomers were supported by electron paramagnetic resonance (EPR) spectroscopy. Based on our results, we propose a mechanism of drug transport by BmrA that is potentially relevant for homologous drug transporters.

**Figure 1:**
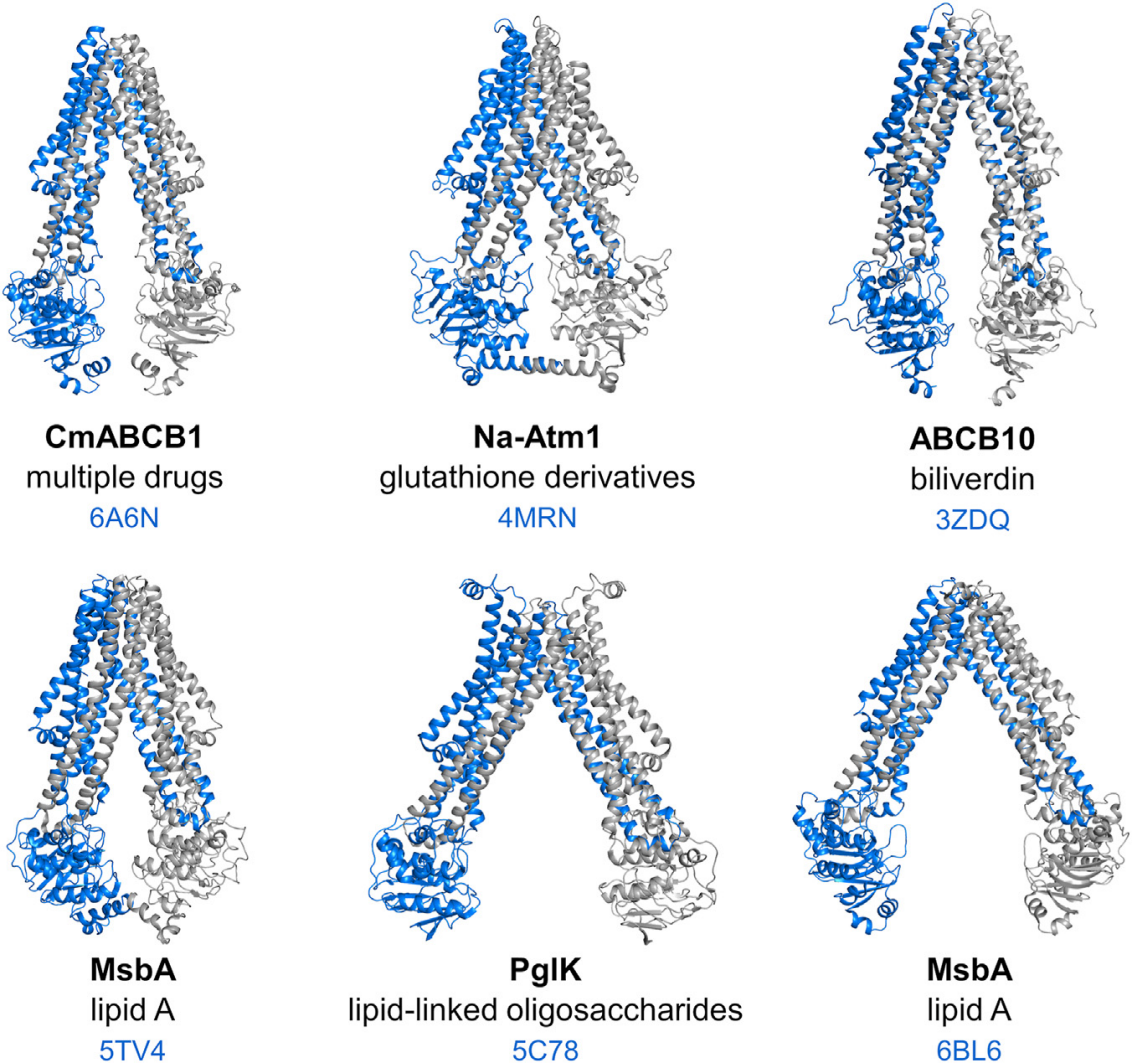
**Inward-facing structures of homodimeric ABC exporters showing diverse degrees of NBD separation.** For each transporter, one monomer is depicted in *blue* and the second in *gray*. PDB codes are listed in *blue*. Two MsbA structures are presented here: one (6BL6) corresponds to a crystal structure obtained with the *Salmonella typhimurium* protein in detergent (60), while the other (5TV4) was solved by cryo-EM from the *Escherichia coli* protein reconstituted in nanodiscs (38). The other structures are described in the following studies (35, 36, 50, 92). NBDs, nucleotide-binding domains; PDB, Protein Data Bank.

## Results

### Engineering a cysteine mutant in the C-terminal region of BmrA

**Figure 2:**
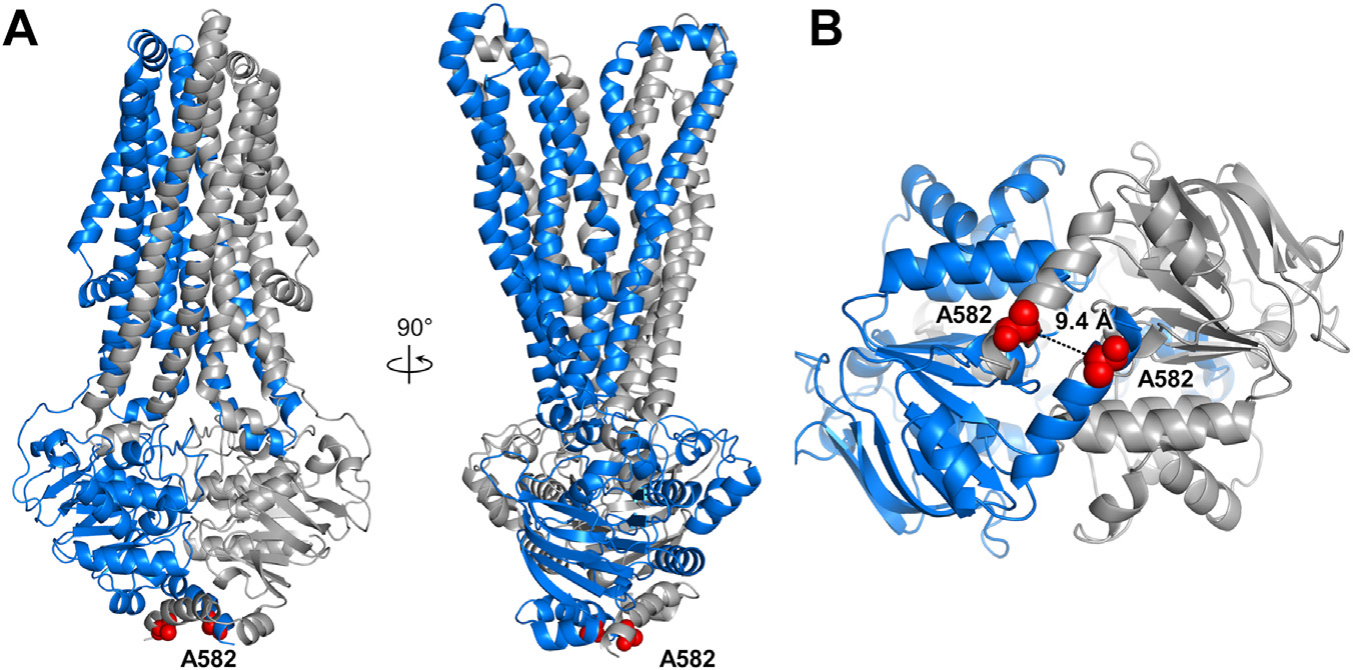
**Outward-facing structure of the homodimeric multidrug ABC transporter BmrA E504A mutant bound to ATP-Mg**^**2+**^. The E504A mutation prevents ATP hydrolysis (93). *A*, side views of BmrA structure (7OW8). The A582 residue near the C-terminal end of the nucleotide-binding domain is shown as *red spheres*. One monomer appears in *blue* and the second one in *gray*. *B*, the bottom view of the nucleotide-binding domains highlights in *red* the proximity of the A582 alpha-carbons, located 9.4 Å apart.

To determine if a large opening between the NBDs of BmrA is required for drug transport, we sought to constrain their separation by introducing a disulfide bond near the C-terminal end of the transporter. We observed on the recent OF structures of BmrA (26), determined either by X-ray crystallography (Protein Data Bank [PDB] 6R72) or cryo-EM (PDB 7OW8), that the A582 residues near the C-terminal end of the NBD are ideally located to generate a disulfide bridge, as they are within 10 Å in the homodimer (Fig. 2). We then introduced the A582C mutation into a cysteine-less mutant of BmrA replacing the single native cysteine at position 436 by a serine. This C436S substitution has been previously shown to have no impact on the transporter activity (27, 28) and is inclusive hereafter when we reference the A582C mutant.

### Probing disulfide bond formation in A582C mutant of BmrA

**Figure 3:**
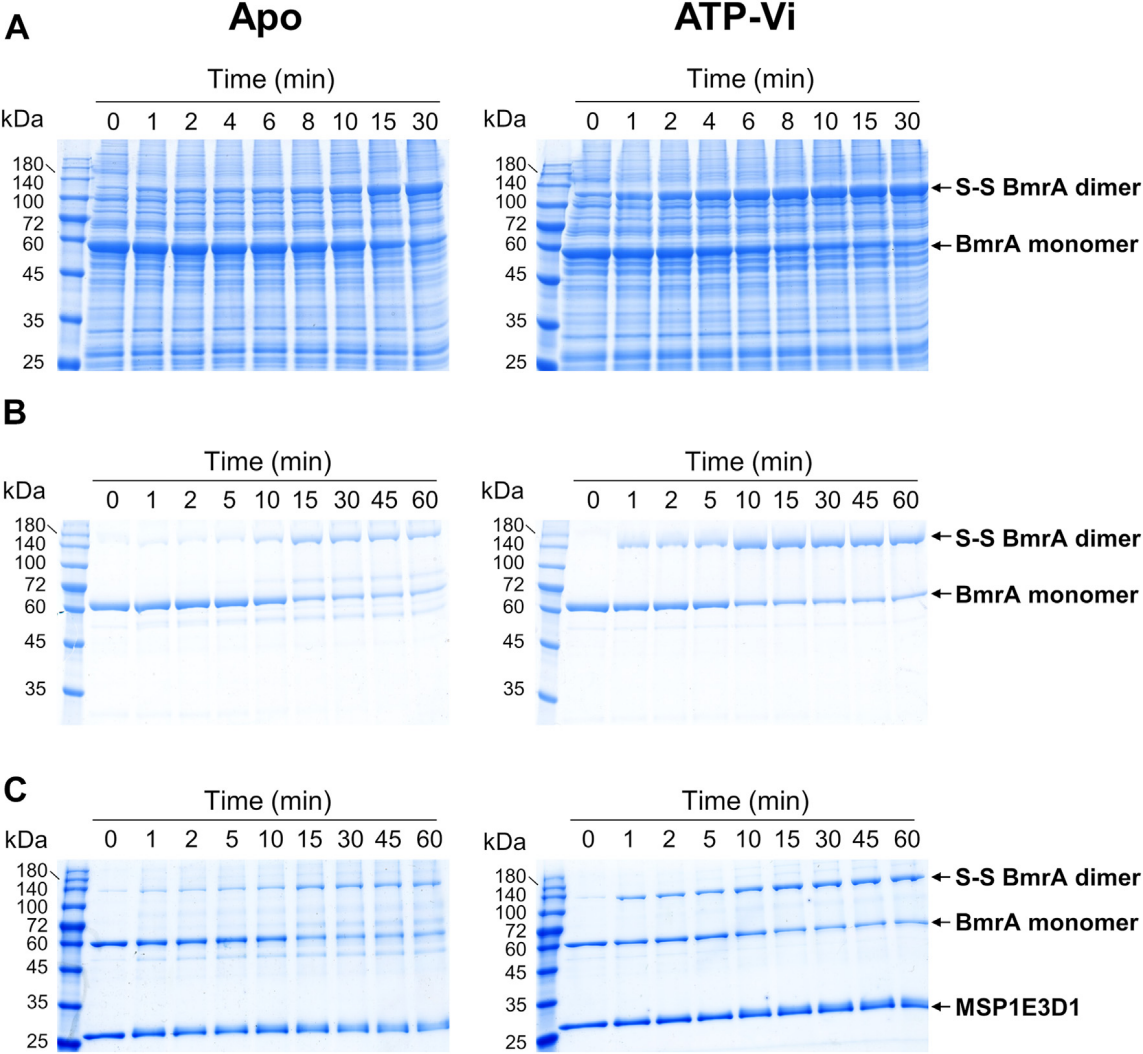
**Kinetics of disulfide cross-linking formation in apo and vanadate-trapped states of BmrA A582C mutant**. Disulfide bond formation was followed at 30 °C in the absence of ligand (*left*) or in the presence of ATP-vanadate (*right*) in inverted-membrane vesicles containing overexpressed BmrA (*A*), or with the protein purified in detergent (*B*) or reconstituted in nanodiscs (*C*). Fractions were analyzed by SDS-PAGE in nonreducing conditions.

We investigated the ability of the A582C BmrA homodimer mutant to form a disulfide bond in different experimental conditions. We first overexpressed the mutant in *E. coli* C41(DE3) strain and prepared inverted membrane vesicles (IMVs) enriched in BmrA (29, 30). These vesicles were incubated in the absence of ligand to favor the IF state of BmrA, or in the presence of ATP/Mg^2+^/Vi to trap the transporter in the OF state (21, 31, 32). Samples were then subjected to mild oxidative treatment. Time-dependent formation of BmrA covalent dimers was analyzed by nonreducing SDS-PAGE. As shown in Figure 3*A*, disulfide cross-link occurred faster after trapping BmrA in the OF conformation. This result suggests that the average distance between the cysteine residues is smaller in the OF state than in the IF state in this native-like membrane environment. Similar results were obtained when the protein was purified in N-dodecyl-β-D-maltoside (DDM)/cholate detergents (26) (Fig. 3*B*) or reconstituted in nanodiscs (33), although the difference was less pronounced in the latter system (Fig. 3*C*).

### Effect of the A582C disulfide bond on the ATPase activity and stability of BmrA

**Figure 4:**
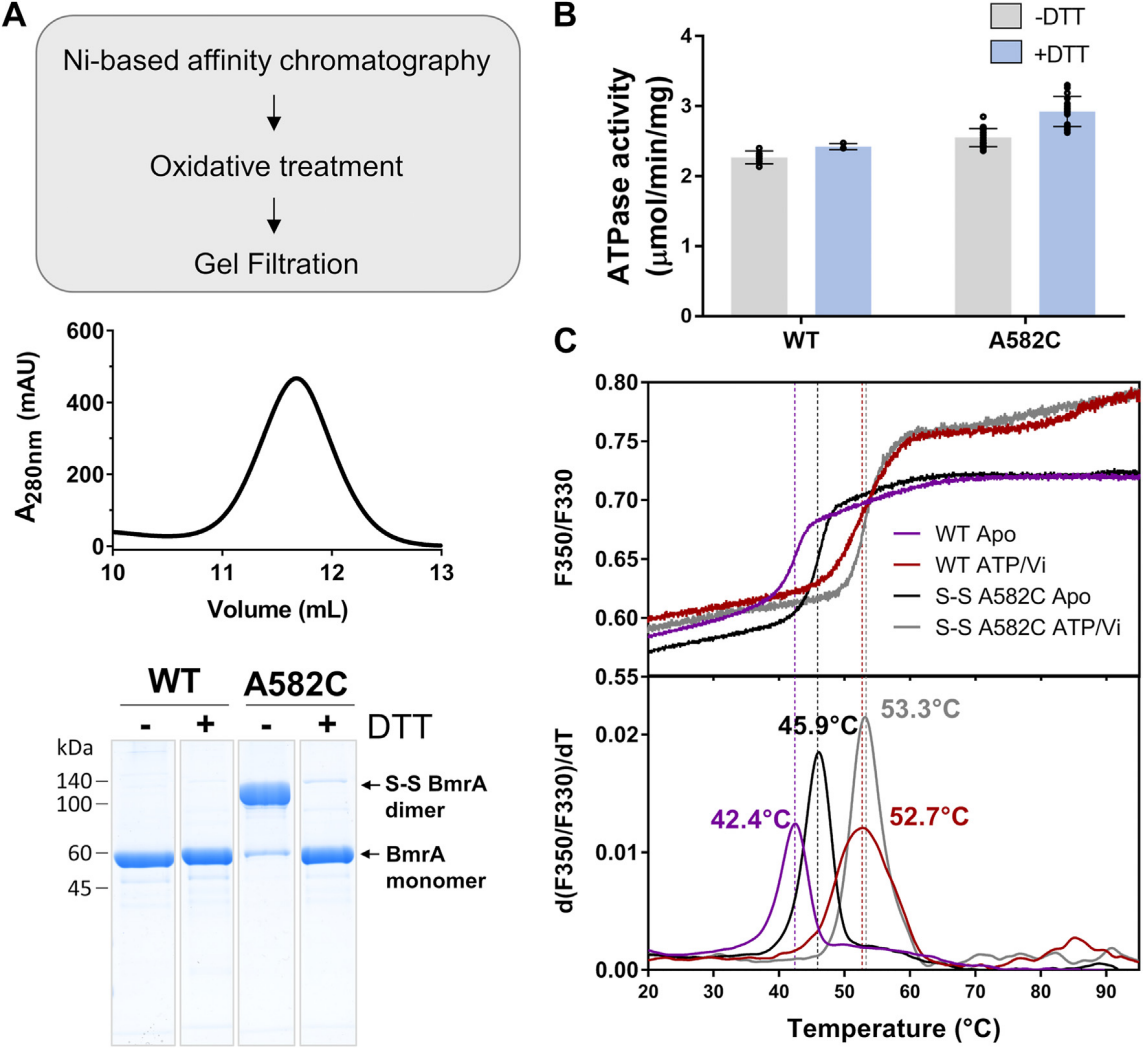
**Purification and biochemical characterization of BmrA A582C mutant.***A*, overview of BmrA mutant purification and oxidative procedure. The purity and quality of detergent-purified protein was assessed by size-exclusion chromatography (for the mutant) and nonreducing SDS-PAGE (for both the WT and the mutant). For clarity, lanes from the same gel were sliced and reordered. *B*, ATPase activities of BmrA WT and A582C mutant in the absence (*gray bars*) or presence (*blue bars*) of reducing agent. Results are shown as mean ± standard deviation of at least two biological replicates (each including technical triplicates). *C*, thermal stability of BmrA WT and A582C mutant in the inward-facing conformation (apo) or trapped in the outward-facing state (ATP-vanadate).

After purification, the A582C mutant was subjected to extensive oxidative treatment in order to form the disulfide bond and the dimer was isolated by size-exclusion chromatography. As shown on a nonreducing SDS-PAGE gel (Fig. 4*A*) more than 90% of the proteins contained the disulfide bond. We next compared the ATPase activities of the cross-linked mutant with the WT protein and found that they were similar both in the absence or presence of DTT (Fig. 4*B*). This showed that the cross-link of the C-terminal end of the NBDs does not impair the ATPase activity of BmrA. Notably the presence of the disulfide bond was not affected by the incubation in the ATPase assay medium (Fig. S1*A*). We also measured the thermal stability of the detergent-purified proteins by differential scanning fluorimetry. In the absence of ligand, the oxidized A582C displayed an increased melting temperature (T_m_) of 3.5 °C than the WT protein (Fig. 4*C*). In contrast, this value was only increased by 0.5 °C in the vanadate-trapped state, suggesting that the disulfide bond mostly increases the stability of the IF state of BmrA.

### Effect of the A582C disulfide bond on BmrA transport

**Figure 5:**
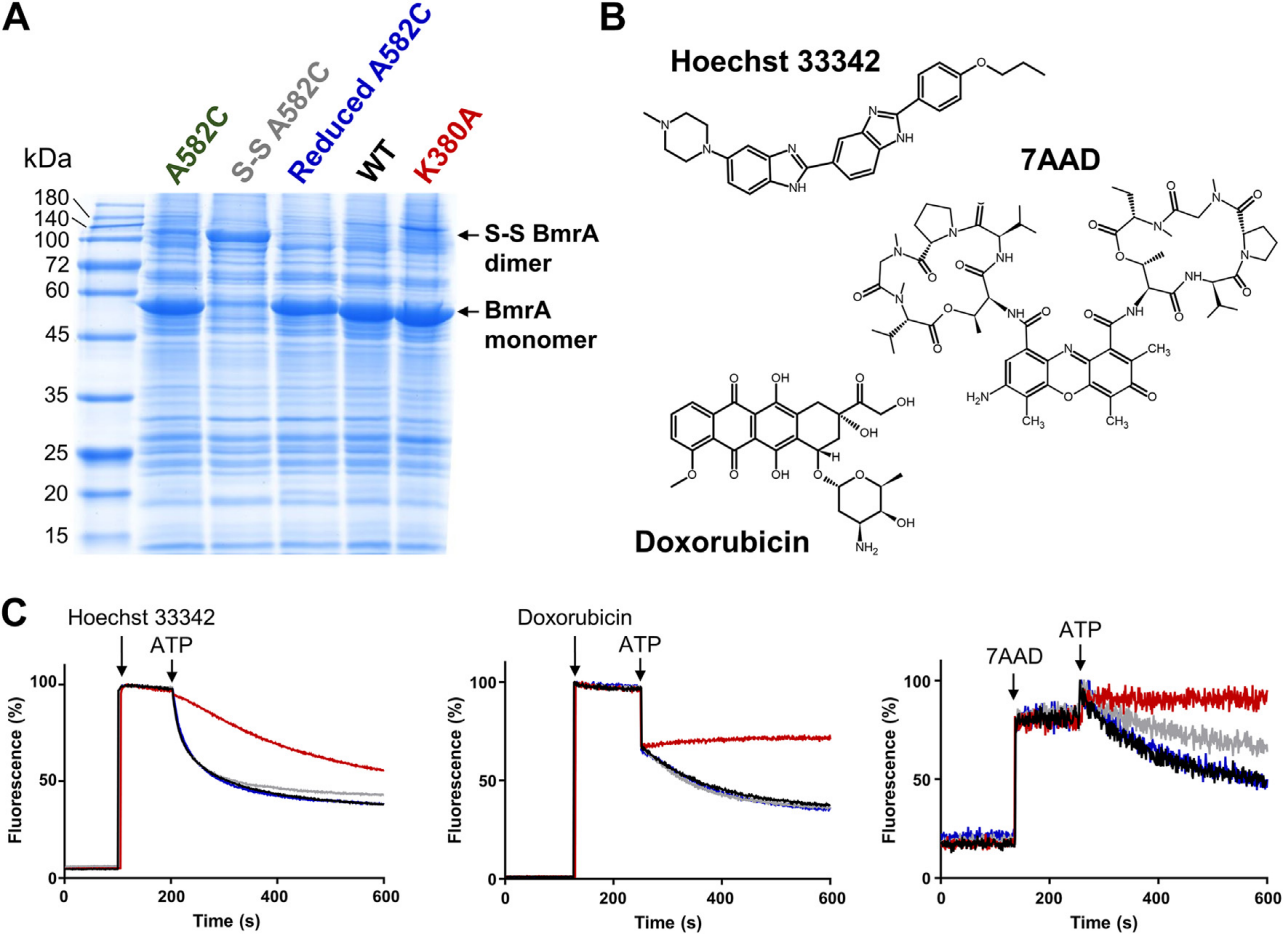
**Drug transport of BmrA A582C mutant overexpressed in *Escherichia coli* inverted membrane vesicles.***A*, SDS-PAGE analysis of inverted membrane vesicles containing nontreated A582C mutant (A582C), treated with an oxidative agent (S-S A582C), or with a reducing agent (reduced A582C). Migration profiles of vesicles with WT and the K380A (inactive mutant) transporters are also shown. *B*, chemical structures of three drugs transported by BmrA. *C*, drug transport assays in inverted membrane vesicles. The colors of the curves correspond to the vesicle samples displayed in the panel A (*black*, WT; *red*, K380A; *gray*, S-S A582C; *blue*, reduced A582C). A representative experiment from at least three repetitions is shown. Drug and nucleotide additions are indicated by an *arrow*.

Next, we evaluated the impact of the A582C disulfide bond on BmrA transport activity in inverted membrane vesicles. As shown by gel electrophoresis the disulfide bridge was present in most of the mutant proteins after the oxidative treatment (Fig. 5*A*). We then checked the ability of the oxidized mutant protein to transport three different drugs (Fig. 5*B*) and compared it to the WT protein and the K380A Walker A inactive mutant (34). Remarkably, the oxidized A582C mutant transports doxorubicin and Hoechst 33342 as efficiently as the WT protein, while retaining ∼60% of 7-amino-actinomycin D transport activity (Fig. 5*C*). In the presence of DTT, the transport of 7-amino-actinomycin D by the A582C mutant was restored to a WT level. In any case, the stability of the disulfide bond was not affected by the transport of the different drugs (Fig. S1*B*). Therefore, this cross-link does not affect the efflux of Hoechst 33342 and doxorubicin, although a larger drug (*i.e.* 7AAD) might require additional flexibility and/or a wider separation of the NBDs to be expelled in an optimal manner.

### Structures of the oxidized A582C mutant and the WT BmrA in the inward-facing state

**Figure 6:**
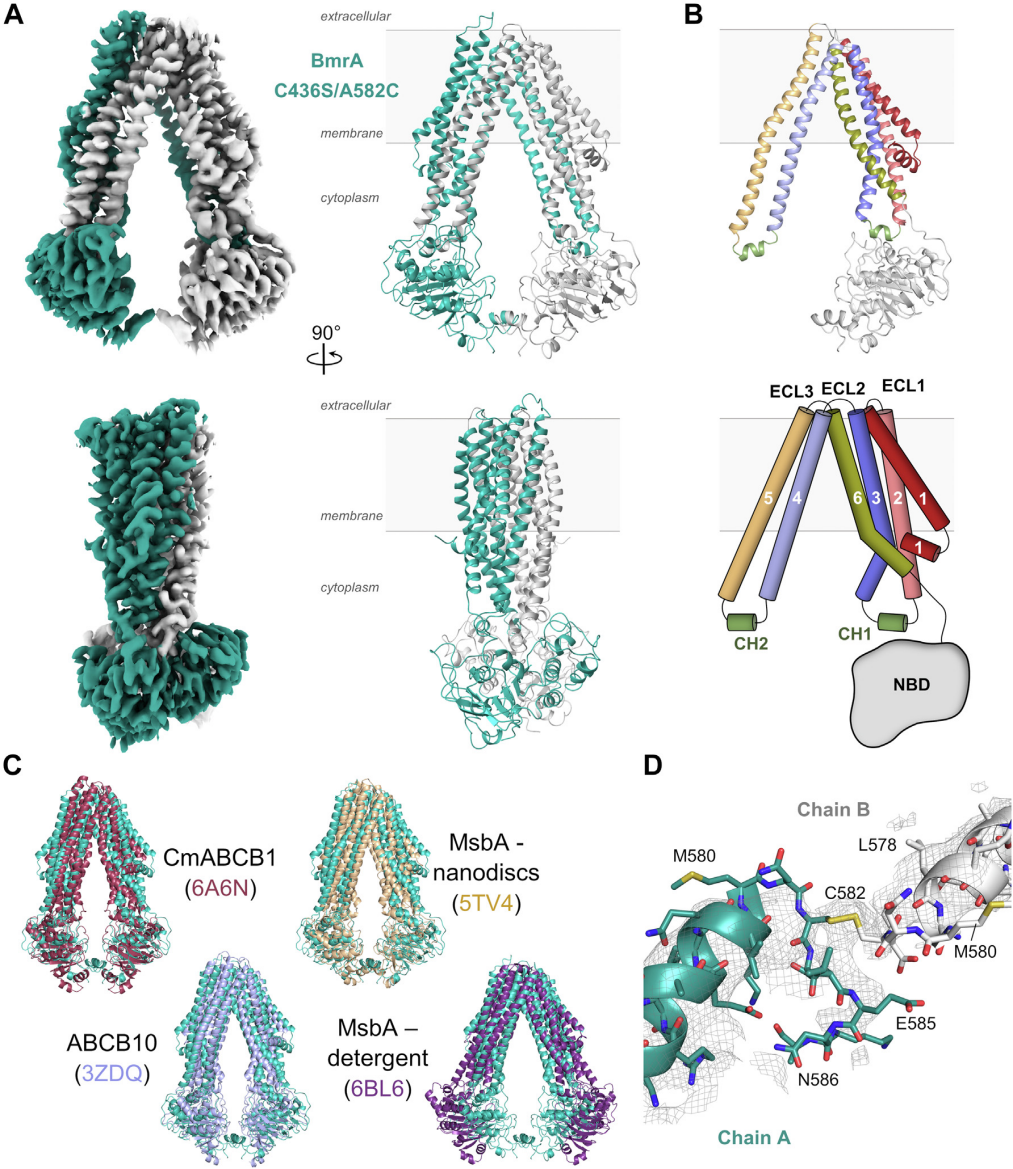
**Cryo-EM structure of BmrA C436S/A582C cross-linked mutant in the inward-facing state.***A*, Cryo-EM density map (*left*) and atomic model (*right*) of BmrA mutant in the inward-facing conformation. The two monomers are colored in *green* and *gray*. A front view is presented on top and the 90° rotated side face is shown underneath. *B*, topology diagram (*bottom*) and representation on the cryo-EM structure (*top*) of BmrA intracellular and membrane regions. *C*, structural alignment of BmrA mutant reported here (*green*) with the apo form of the eukaryotic CmABCB1 transporter (6A6N, *red*), the mitochondrial transporter ABCB10 (3ZDQ, *blue*), the nanodisc-embedded MsbA cryo-EM structure (5TV4, *yellow*) or the crystal structure of purified-detergent MsbA (6BL6, *purple*). *D*, close-up view of BmrA C436S/A582C structure showing electron density for the C582 residues forming a disulfide bridge. ECL, extracellular loop; CH, coupling helix; NBD, nucleotide-binding domain.

**Figure 7:**
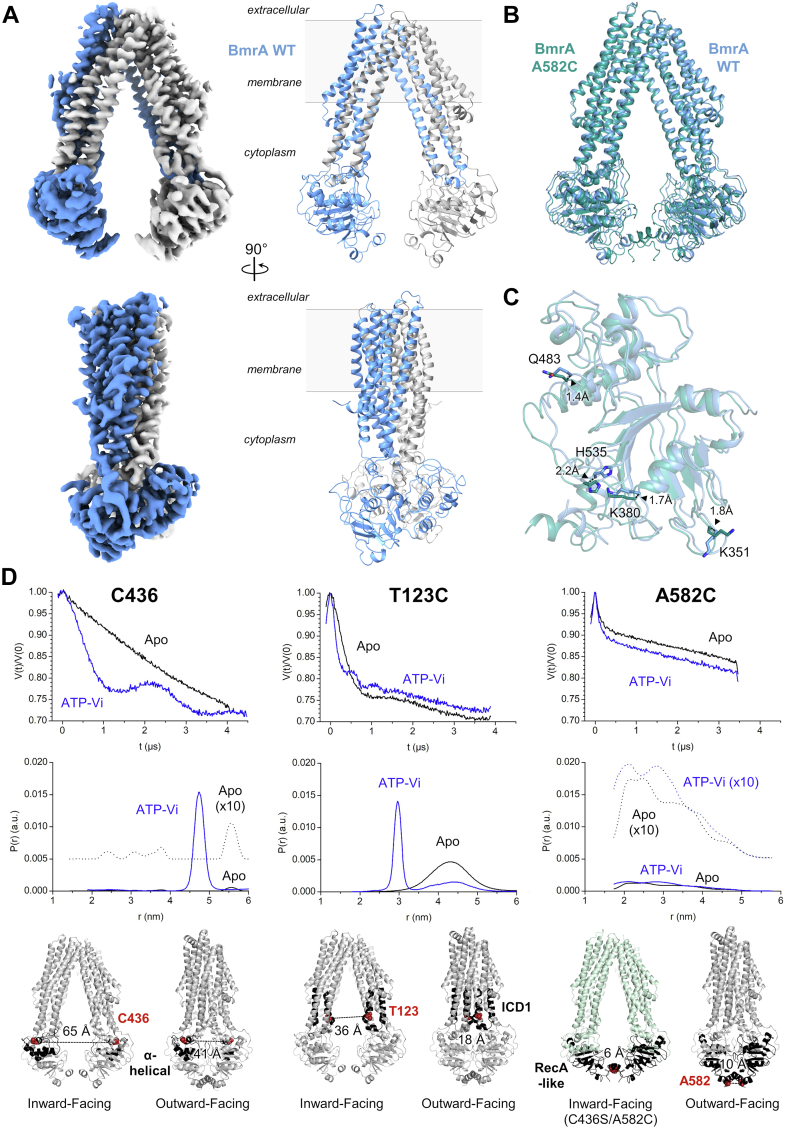
**Cryo-EM structure of WT BmrA in the inward-facing state and distance distributions in the IF and OF states by DEER experiments.***A*, Cryo-EM density map (*left*) and atomic model (*right*) of WT BmrA. The two monomers are colored in *blue* and *gray*. A front view is presented on top and the 90° rotated side face is shown underneath. *B*, alignment of BmrA WT (*blue*) and BmrA C436S/A582C mutant (*green*) structures showing high similarity. *C*, close-up view on the NBD of the alignment presented in panel (*B*). The distance separating the same residue in each structure is indicated. *D*, distance distributions in BmrA WT (*left*), T123C (*middle*) and A582C (*right*) from Q-band DEER experiments. For T123C and A582C spin labeling, the naturally present Cys436 was also mutated into a serine (C436S mutant). Data were recorded on the protein apo form (IF, *black* traces) or in presence of ATP-vanadate (OF, *blue* traces). Where indicated a ten time magnification is also shown. Q-band DEER traces are presented in Figs. S6–S8. Residues used for spin labeling are shown underneath as red spheres on the IF (8QOE) and OF (7OW8) structures of BmrA. This excludes residue at position 582 which is absent in the IF structure (8QOE) and thus shown on the C436S/A582C cross-linked mutant (8CHB, *green*). The α-helical, ICD1 or RecA-like subdomains are in *black*. DEER, double electron-electron resonance; ICD, intracellular domain; IF, inward-facing; NBDs, nucleotide-binding domains; OF, outward-facing.

As the A582C disulfide cross-link increased the stability of BmrA without major impact on its function, we aimed to solve its structure to understand the basis for its preserved functionality. We determined by cryo-EM the structure of the oxidized A582C mutant at 3.14 Å resolution. Cryo-EM data collection and refinement statistics are presented in Figs. S2, S3, and Table S1. The corresponding map and model are presented in Figure 6, *A* and *B* (PDB 8CHB). Consistent with its ability to transport drugs, the mutant displays a canonical IF conformation similar to that seen in related multidrug ABC transporters from the type IV subfamily (17). These include the apo structures of the eukaryotic drug transporter CmABCB1 (35), the biliverdin mitochondrial transporter ABCB10 (36, 37), and the *E. coli* lipid A carrier MsbA reconstituted in nanodiscs (38) (Fig. 6*C*). This structure agrees with results from a recent small-angle neutron scattering study of WT BmrA (31), where the degree of NBD separation is intermediate between the largest opening observed in MsbA (Fig. 6*C*), and the closed state in the OF structure of BmrA (PDB 6BL6 and PDB 7OW8, respectively). Interestingly a clear density bridging the two monomers is visible at the lower part of the two NBDs. This allows us to build the disulfide bond between the two Cys582 residues (Fig. 6*D*). In order to rule out the possibility that the disulfide bond restrained a wider opening of BmrA, we also determined by cryo-EM the structure of the WT protein in the resting state at a resolution of 3.16 Å. Cryo-EM data collection and refinement statistics are listed in Figs. S4, S5, and Table S1. The map and model of WT BmrA are presented on Figure 7*A* (PDB 8QOE). The C-terminal domain of the transporter appears more flexible than the rest of the protein, and residues 578 onward could not be modeled. As shown in Figure 7*B*, and on Movie S1, the structure of WT BmrA is highly similar to that of the A582C cross-linked mutant (rmsd of 0.86 Å over 931 residues). Both structures adopt an IF conformation where the whole NBDs are moderately separated. The transmembrane helices are perfectly superposed (rmsd of 0.45 Å over 549 residues) while the NBDs are slightly less separated in the mutant structure. However this difference is minimal and represents only 1.4 to 2.2 Å when comparing the C_α_ position of residues K351, K380, Q483, and H535 from the NBD conserved motifs (A-loop, Walker A motif, ABC signature, and H-loop, respectively) between the WT and the mutant proteins (Fig. 7*C*). We also observed some flexibility of the transporter, especially the NBDs slightly opening and closing when comparing the different classes of the WT and the C436S/A582C mutant cryo-EM refinement (Figs. S2 and S4). Movie S2 shows the molecular morph from the most closed to the most opened classes, highlighting this flexibility. A similar amplitude of opening can be seen in the C436S/A582C mutant and in the WT datasets, underlining the similitude of the two structures. As both were solved at identical resolution, it is presumably the cross-link bridge between the two monomers that rigidifies the C-terminal region of the mutant transporter. This allows us to build an additional ten residues in the oxidized C436S/A582C mutant structure.

### Site-directed spin labeling and EPR spectroscopy to investigate the degree of NBD separation

To further validate the distance separation between the NBDs in the apo structure, and to study the transition from the IF to OF conformations, we introduced spin labels in each monomer of the WT transporter and measured the distance between the two nitroxide molecules by pulsed EPR spectroscopy. We first used the native cysteine (C436) localized in the α-helical subdomain of the WT protein. Distance distribution variations were analyzed in the absence of ligand (apo, IF state) and after vanadate-induced ADP trapping (ATP/Vi, OF state). In the absence of ligand, the distance between the spins was outside of measurable range (>60 Å; Figs. 7*D* and S6), consistent with the BmrA IF cryo-EM structure where the C_α_ of the two cysteines are separated by 65 Å. In the presence of ATP/Vi, clear modulations appeared on the double electron-electron resonance (DEER) trace leading to a narrow distance distribution around 47 Å. This value is similar to the 41 Å distance separating the two C_α_ of C436 residues in the BmrA OF structure (7OW8). We also placed the spin label at position 123 in intracellular domain (ICD1) of BmrA to measure distance distributions within the range of the DEER experiments. To this aim, we engineered the C436S/T123C double mutant by site-directed mutagenesis. In the absence of ligand, a broad distance distribution between the labels was visible and centered around 43 Å, while in the presence of ATP/Vi, it was mostly converted to a narrow distribution at 30 Å (Figs. 7*D* and S7). Considering the size and conformational landscape of the nitroxide label, both measurements are consistent with the IF and OF structures of BmrA where the C_α_ of T123 are separated by 36 and 18 Å respectively. Because the ICD1 always remains in contact with the NBD, these results indicate that a large distance variation occurs in the N-terminal part of the NBD when the transporter switches from the IF to the OF states. By contrast, labeling at position 582 in the C436S/A582C mutant did not lead to any significant changes between the apo and the ATP/Vi bound forms where broad distributions in the 20 to 40 Å distance range are observed in both cases (Figs. 7*D* and S8). This result is consistent with a limited movement of the lower part of the NBDs upon the transition between the IF and OF conformations of the transporter. Altogether the distances measured show that a large variation occurs in the ICD1 and in the α-helical subdomain of the NBD when the transporter switches from the IF to the OF states, in contrast to the NBD C-terminal part which undergoes a limited motion.

### A tweezers-like motion for the NBDs of BmrA

**Figure 8:**
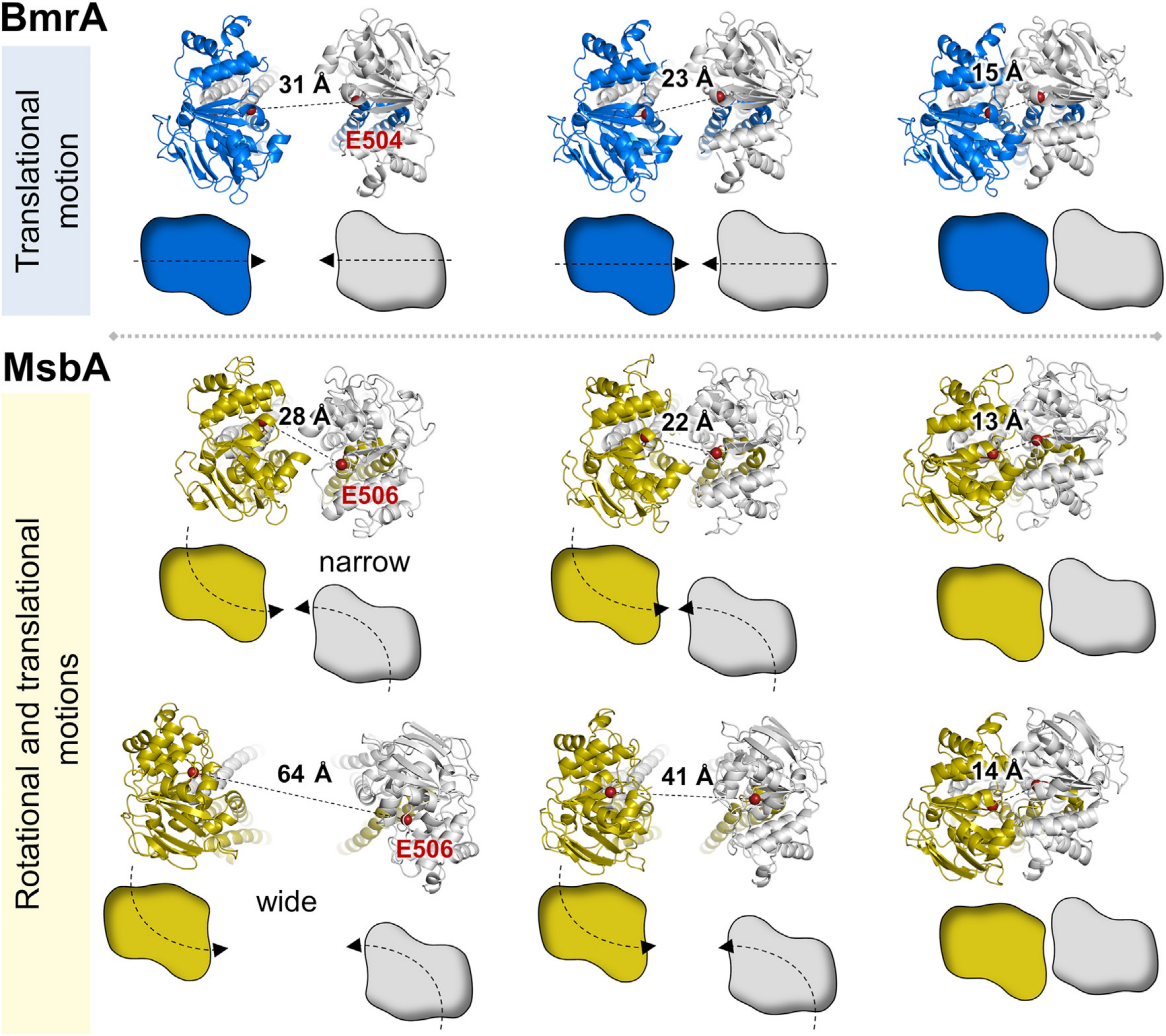
**Tweezers-like mechanism of NBD closure in BmrA and comparison with MsbA.** Snapshots of BmrA (*top*), MsbA narrow (*middle*) or MsbA wide (*bottom*) transiting from the IF (8QOE for BmrA, 5TV4 for MsbA *narrow*, and 6BL6 for MsbA *wide open*) to the OF conformation (7OW8 for BmrA, 5TTP for MsbA narrow and 3B5Z for MsbA *wide*). The *middle panels* correspond to the midpoint of the IF to OF transition molecular morph. The C_α_ of the two catalytic glutamate residues is shown by a *red sphere* at different stages of the closing motion. A scheme representing the differences in the NBD closing motion between BmrA (single translation) and MsbA (simultaneous rotation and translation) during the dimerization is shown underneath each structure pose. IF, inward-facing; NBD, nucleotide-binding domain; OF, outward-facing.

By generating a molecular morph between the IF and OF conformations of WT BmrA, we found that the NBDs of the transporter can function according to a tweezers-like motion. The NBDs of BmrA are well aligned in the apo state and, following ATP binding, they dimerize in a straight manner (Fig. 8 and Movie S3), mimicking a tweezers movement similar to the mechanism previously proposed for the MalK subunits of the maltose importer (39). This mechanism is distinct from what was observed for the lipid A exporter MsbA, which shares a substantial sequence homology with BmrA (30% identity and 26% of strong similarity). In MsbA, two motions bring the two NBDs into alignment (Fig. 8, Movies S3 and S4), ultimately placing the Walker A motif of one monomer in proximity to the ABC signature (LSGGQ motif) of the other (38, 40). These motions include a hinge rotation at the extracellular side to bring the NBDs closer, and a translation movement leading to their dimerization (40). This is visible for the narrow MsbA structure obtained in nanodiscs which presents the same degree of NBD opening as BmrA and for the wide, largely open structure of MsbA obtained in detergent (Fig. 8), confirming that the mechanism of NBD closure is intrinsic to the protein. In comparison, the NBDs of BmrA monomers are well aligned and dimerize in a single translation (Fig. 8 and Movie S3). This movement includes a rotation of the α-helical subdomain relative to the RecA-like subdomain (or reciprocally) (Fig. S9). During this rotation, the family signature motif moves into a favorable position to interact with the nucleotide across the dimer interface. Because the coupling helices of the TMDs are docked into a cleft at the interface between these subdomains, this movement is also critical for interdomain communication in ABC transporters (35, 41, 42, 43). The fact that the disulfide bond at position 582 did not prevent this movement (Fig. S9) is consistent with the preserved functionality of the cross-linked mutant.

## Discussion

**Figure 9:**
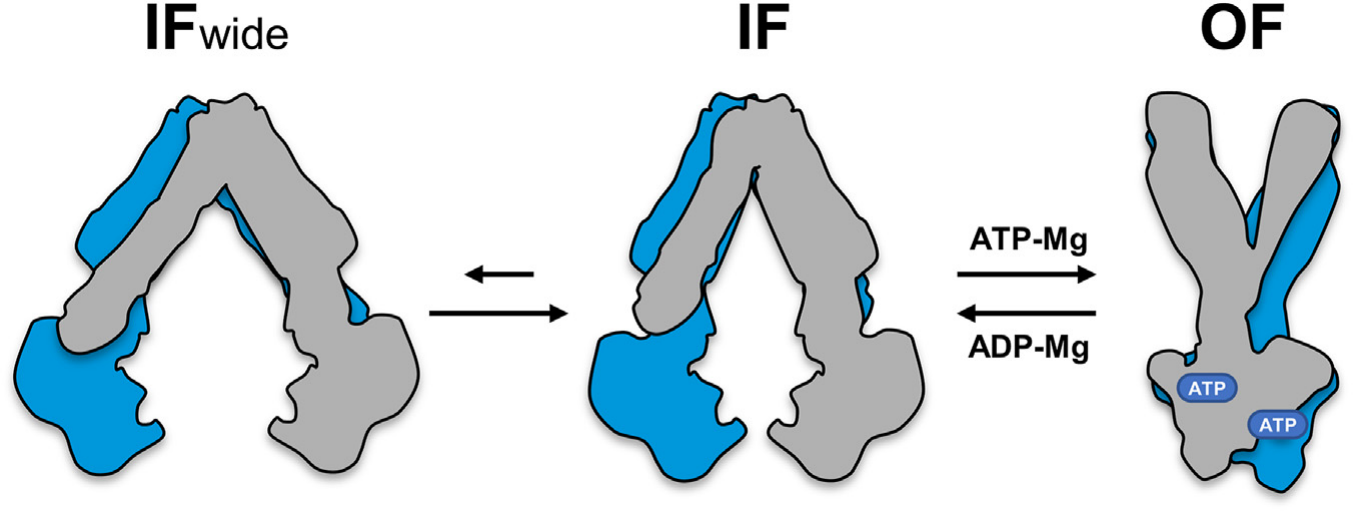
**Proposed transport cycle of BmrA.** In the apo state BmrA is predominantly inward-facing where the C-terminal parts of the NBDs are in close proximity. In the presence of ATP the NBDs dimerize following a tweezers-like motion and the transporter switches to an outward-facing conformation. Nevertheless, the transporter may occasionally display a wider inward-facing state to possibly accommodate larger drugs. NBDs, nucleotide-binding domains.

ABC transporters are powered by their NBDs which use the energy of ATP binding and hydrolysis to drive the alternating access of the substrate-binding site located in the TMDs. Reciprocally, the ATPase activity of ABC proteins is generally stimulated in the presence of substrates, although the degree of stimulation varies depending on the transporter (44). Despite extensive work on exporters in the last few decades, their catalytic and transport cycles are still far from being understood (17, 45, 46). A subject of debate is the extent of NBD separation in ABC transporters, which differentiates the ATP-switch transport model (47) from the constant-contact model (48, 49). In addition, several apo structures of ABC exporters, such as the lipid A transporter MsbA which adopts a widely open IF state with the NBDs far apart (40), have been generally considered nonphysiological due to the presence of detergent or possible crystallization artifacts (17). Apart from MsbA, a second widely open structure was reported for the PglK ABC transporter (Fig. 1), and the relevance of this structure was also questioned (50). Hence why numerous studies have deployed an arsenal of biophysical techniques such as EPR spectroscopy (22, 51, 52, 53, 54), luminescence resonance energy transfer (55, 56, 57) and single-molecule fluorescence resonance energy transfer (58, 59) to measure the distance separation between the NBDs of ABC transporters in different detergents or lipidic environments. A spin-labeled nanobody was recently used to investigate the conformational cycle of MsbA by EPR in various environments, including in *E. coli* cells. Unexpectedly, the wide open IF conformation of MsbA was found to be prominently populated in *E. coli* cells (18). In this study MsbA displayed a similar conformation in IMVs, detergent and liposomes, while the protein predominantly adopts a narrow IF conformation in the absence of nucleotides when reconstituted into nanodiscs. This suggested that the physical constraint imposed by these nanostructures restricts the amplitude of the NBD separation. It has been proposed that the wide IF conformation of MsbA is necessary to sufficiently open the lateral portals in order to grant access of the large lipid A molecule to the substrate-binding cavity (18, 60). Here, we investigated whether a wide separation of the NBDs is required for the proper function of the bacterial multidrug ABC transporter BmrA. We therefore engineered a A582C mutation near the C-terminal end of the NBD to prevent the physical separation of the NBDs by cross-linking, and analyzed the consequences of the disulfide bridge on the protein activity. A similar strategy was used with the eukaryotic P-glycoprotein (P-gp or ABCB1) (61). This study showed that the cross-linked P-gp retained substantial drug-stimulated ATPase activity for the majority of the drugs tested, suggesting that the C-terminal ends of the two NBDs do not undergo substantial motion relative to each other during the catalytic cycle. However, little to no ATPase activation was observed in the presence of vinblastine and colchicine. This suggests that binding and/or ATPase stimulation of some drugs requires additional motion of the TMDs and/or NBDs, as compared to the cross-linked protein (61). Drug transport was unfortunately not assayed in that work. Here, we addressed whether a full separation of the NBDs is necessary to support multidrug transport in the bacterial ABC transporter BmrA. We found that the presence of the disulfide bond between the NBDs did not prevent the ATPase activity nor the transport of drugs, demonstrating that a large separation of the NBDs is not mandatory for the transport function. While the presence of the disulfide bond in BmrA A582C fully supported the Hoechst 33342 and doxorubicin export, the transport of the larger molecule 7-amino-actinomycin D was however less effective as compared to the WT or the mutant protein in reducing conditions. This suggests that the optimal binding and/or transport of some molecules may require a larger degree of freedom or wider motion of BmrA as compared to the cross-linked transporter (Fig. 9). A previous low-resolution cryo-EM study performed in lipidic bilayer reported two IF conformations of BmrA where most molecules adopt an intermediate NBD opening, while a subset presented a more open state (62). A high plasticity is certainly an essential feature of multidrug ABC transporters (see for reviews (10, 44)). According to numerous biochemical and biophysical experiments, it is evident that for a majority of type IV ABC transporters (5), or type I ABC exporters, the IF apo state is flexible (19, 21, 22, 63). While the OF state in which the NBDs are tightly closed is mostly rigid (31, 32, 64), some flexibility in the external region of the transmembrane helices were identified, notably in the helices TM1 and 2 (26, 65) and an extracellular gate identified in the TM287/288 transporter (66). This local flexibility at the extracellular side of these transporters may contribute to drug release. The plasticity of multidrug ABC transporters permits the exploration of multiple states, where the opening and closing motions of the NBDs are directly translated to the transmembrane helices and remodeling of the drug-binding pocket. This may contribute accommodating a larger variety of substrates (23). Mirroring this, minor structural differences between substrate- and inhibitor-bound ABCB1 sites appeared amplified toward the NBD, suggesting how the plasticity of the drug-binding site controls the dynamics of the NBDs (67). The restriction of the separation of BmrA NBDs by a disulfide cross-link clearly supported drug transport activity, even if the catalytic domains can only undergo a limited separation. Although BmrA is a homodimer protein, it should be noted that heterodimeric multidrug transporters, such as EfrCD (68), TM287/288 (69, 70), or TmrAB (71, 72) have not been observed with a wide open conformation and often retained their C-terminal helices in close proximity. This suggests that a wide separation of the NBDs in multidrug transporters is not essential for the transport of most substrates as demonstrated here for BmrA. Finally, we solved the first IF structure of BmrA, providing not only a structural framework for further experimental studies but also complementing the previously published OF structures of BmrA (26) to further understand the molecular details of the alternating access transport mechanism. From the comparison between the IF and OF structures of BmrA, this mechanism appears clearly different from the one of MsbA since the separation and alignment of their respective NBDs greatly differ. In contrast to MsbA the NBDs of BmrA can function with a tweezers-like motion similar to the originally proposed mechanism for the maltose ABC transporter (39, 73). Furthermore, the OF structure of MsbA (PDB 3B5Z) showed a wide opening at the level of the outer leaflet (40), suggesting a floppase-type mechanism, in which lipid A is moved from the inner layer to the outer layer of the membrane. In contrast to this, the OF structure of BmrA has limited space for the substrates to exit at the outer-leaflet of the membrane (Movie S4). This may imply that hydrophobic substrates of BmrA are transported to the extracellular space rather than into the lipid bilayer, as suggested for the P-gp homologue, CmABCB1 (35, 74), and in contrast to the flippase mechanism proposed for the fungal PDR5 transporter (75). Deciphering the molecular mechanism operating in various multidrug ABC transporters will require a combination of biochemical and biophysical methodologies to illuminate our understanding of their functioning mechanisms.

## Experimental procedures

### Site-directed mutagenesis

BmrA C436S/A582C and C436S/T123C mutants were generated by introducing a cysteine at position 582 or 123 respectively into a Cys-less (C436S) hexa histidine-tagged pET23 BmrA using the QuikChange Lightning Site-Directed Mutagenesis Kit (Agilent Technologies). The following primers were designed using QuikChange Primer Design software (https://www.agilent.com/store/primerDesignProgram.jsp) and used for the mutagenesis: tccgggacaatcagagaaaatatttcctacgggttagaac (C436S Forward), gttctaacccgtaggaaatattttctctgattgtcccgga (C436S Reverse), tgaacagcagctgaaaatgaattgcgacttagaaaacaaagccggg (A582C Forward), cccggctttgttttctaagtcgcaattcattttcagctgctgttca (A582C Reverse) ttatttcgatacaaatgcttccggagaatgcgtcagccgtgtaacg (T123C Forward), and cgttacacggctgacgcattctccggaagcatttgtatcgaaataa (T123C Reverse).

### BmrA overexpression

Expression of BmrA in C41(DE3) *E. coli* strain (76) was performed as described (77) with minor modifications. A freshly transformed colony was inoculated in 100 ml LB containing 100 μg/ml of ampicillin and grown overnight at 37 °C with shaking at 180 rpm. The overnight preculture was then used to inoculate 1 L of 2xYT medium containing 100 μg/ml ampicillin to an optical density (*A*_600 nm_) of 0.05. The flask was then incubated à 37 °C with shaking at 180 rpm until *A*_600 nm_ reached 0.6. Protein expression was induced by addition of 0.7 mM IPTG. After 4 h at 25 °C, cells were harvested by centrifugation at 6000*g* for 15 min at 4 °C and the bacterial pellet was stored at −80 °C.

### IMVs preparation

The BmrA IMVs were prepared as previously described (25, 30). In short, bacterial pellets were resuspended in 50 mM Tris–HCl pH 8.0, 5 mM MgCl_2_, 1 mM DTT, and 5 μg/ml Dnase I. Cells were then lysed by three passages at 18,000 psi through a Microfluidizer. Unbroken cells were then removed by centrifugation at 15,000*g* for 30 min at 4 °C before collecting the membrane fraction by ultracentrifugation for 1 h at 150,000*g* at 4 °C. Pellets were resuspended in 50 mM Tris–HCl pH 8.0, 1.5 mM EDTA, 1 mM DTT and ultracentrifuged again for 1 h at the same speed. Final membrane vesicles were resuspended in 20 mM Tris–HCl pH 8.0, 1 mM EDTA and 300 mM sucrose (typically 2 ml per L of culture) then flash-frozen in liquid nitrogen and conserved at −80 °C. Total protein concentration in IMVs was measured by bicinchoninic acid assay, and BmrA overexpression was analyzed on SDS-PAGE stained with Coomassie brilliant blue.

### Purification of BmrA WT and C436S/T123C mutant

BmrA-enriched IMVs were solubilized at 2 mg/ml in 50 mM Tris–HCl pH 8.0, 100 mM NaCl, protease inhibitor cocktail, 10% glycerol, and 1% DDM. After 1 h of solubilization under gentle agitation at 4 °C, IMVs were ultracentrifuged 1 h at 150,000*g* at 4 °C. The supernatant was loaded onto a Ni-NTA column equilibrated with 50 mM Tris–HCl pH 8.0, 100 mM NaCl, 10% glycerol, 0.0675% DDM, 0.04% sodium cholate and 10 mM imidazole. The resin was first washed with five column volumes of 50 mM Tris–HCl pH 8.0, 500 mM NaCl, 10% glycerol, 0.0675% DDM, 0.04% sodium cholate and then with 20 column volumes of 50 mM Tris–HCl pH 8.0, 100 mM NaCl, 10% glycerol, 0.0675% DDM, 0.04% sodium cholate, and 20 mM imidazole. Bound proteins were eluted with an imidazole gradient from 20 to 500 mM. The fractions corresponding to the peak of elution were then pooled and dialyzed (cutoff 12–14 kDa) overnight, and again for 4 h the next day, against 50 mM Hepes-NaOH, 50 mM NaCl, 10% glycerol, 0.035% DDM, and 0.03% sodium cholate at 4 °C. The protein concentration was then determined from UV absorbance at 280 nm by using Nanodrop spectrophotometer, and BmrA molar extinction coefficient (ε_280nm_ = 38,850 M^−1^ cm^−1^). The protein was then concentrated to 3 to 7 mg/ml on 100 kDa cutoff Amicon Ultra-15 device at 1000*g* at 4 °C before being stored at −80 °C.

### Preparation of disulfide cross-linked C436S/A582C mutant of BmrA

The mutant protein was purified as previously described for BmrA WT and C436S/T123C variant, except that the eluted protein fractions were pooled and subjected to an oxidative treatment with 0.5 mM copper(II) sulfate and 1.5 mM 1,10-phenanthroline in order to promote the formation of a disulfide bond between the two BmrA monomers. The sample was then concentrated to 10 mg/ml on 100 kDa cutoff Amicon Ultra-15 device at 1000*g* at 4 °C, before being loaded onto Superdex 200 increase 10_300 Gl using 50 mM Hepes/NaOH pH 8.0, 10% glycerol, 50 mM NaCl, 0.035% DDM, and 0.03% sodium cholate as mobile phase. Fractions corresponding to the peak of elution were then pooled and the protein was either stored at 4 °C for cryo-EM analysis or at −80 °C for biochemical characterization. The protein concentration was determined from UV absorbance at 280 nm by using Nanodrop spectrophotometer.

### Transport assays

Transport of the three fluorescent drugs was measured in IMVs using a Quanta Master I fluorometer (Photon Technology International) as previously described (25). The excitation and emission wavelengths were respectively set at 355 and 457 nm for Hoechst 33342, 546 and 647 nm for 7-amino-actinomycin D, and 480 and 590 nm for doxorubicin, with spectral bandwidths of 2 and 4 nm for excitation and emission, respectively. Experiments were performed in a quartz cuvette with a final volume of 1 ml in 50 mM Hepes/KOH pH 8.0, 8.5 mM NaCl, 4 mM phosphoenolpyruvate, 60 μg/ml pyruvate kinase (Roche), and 2 mM MgCl_2_. Either 100 μg (for doxorubicin transport) or 200 μg (for Hoechst 33342 or 7-amino-actinomycin D) of IMVs were added and the fluorescence was recorded for 2 min before addition of the fluorescent drug (2 μM of Hoechst 33342 or 10 μM of 7-amino-actinomycin D or doxorubicin). After 2 min, transport was initiated by the addition of 2 mM ATP and monitored for at least 10 min.

### ATPase activity assays

The ATPase activity of detergent-purified BmrA was essentially measured as described (25) with minor modifications. Activities of ATP hydrolysis were measured in a total volume of 700 μl in 50 mM Hepes/KOH pH 8.0, 10 mM MgCl_2_, 4 mM phosphoenolpyruvate, 0.3 mM NADH, 32 μg/ml of lactate dehydrogenase, 60 μg/ml pyruvate kinase, 10 mM ATP, 0.035% DDM, and 0.03% sodium cholate. The buffer was heated at 37 °C for 5 min before adding 3 μg of purified protein. Activity of BmrA was then measured by following the NADH absorbance at 340 nm during 20 min at 37 °C.

### Thermal denaturation assays

The thermal denaturation of detergent-purified BmrA mutant was performed by nano-differential-scanning fluorimetry using a Prometheus NT.48 instrument (NanoTemper technologies), as previously described (78). All BmrA samples were at 1 mg/ml and where specified supplemented with 10 mM ATP, 10 mM MgCl_2_, and 1 mM sodium orthovanadate (Vi). Samples were incubated for 20 min at room temperature after the addition of ligands. The capillaries were then filled with 10 μl of the sample mixture and placed on the sample holder. A temperature gradient of 1 °C/min from 25 to 95 °C was applied and the intrinsic protein fluorescence was recorded at 330 and 350 nm. The ratio of fluorescence intensities at 350/330 nm was used to determine the melting temperature T_m_.

### Disulfide bond formation kinetics

Kinetics of disulfide cross-linking between the two BmrA monomers was followed in different conditions (apo and ATP/Mg^2+^/Vi) and environments (IMVs, detergent, nanodiscs).

#### Inverted membrane vesicles

For the protein overexpressed in *E. coli*, IMVs were diluted at 1.3 mg/ml in 50 mM Hepes/KOH pH 8.0, 50 mM NaCl, and 5 mM MgCl_2_, supplemented with 4 mM ATP, and 0.2 mM sodium orthovanadate (Vi) where specified. After 20 min of incubation at 30 °C, oxidation was initiated by addition of 6.25 μM copper(II) sulfate and 18.75 μM 1,10-phenanthroline. At 0, 1, 2, 4, 6, 8, 10, 15, and 30 min time points, 16 μl samples (20 μg BmrA) were then withdrawn from the reaction. For each sample 10 mM of N-ethylmaleimide (NEM) were then immediately added to stop the reaction. Proteins were analyzed by SDS-PAGE in non-reducing conditions.

#### Detergent

For the protein purified in detergent, samples at 1.6 mg/ml were first reduced with 3.5 mM DTT for 10 min at 25 °C. Reduced proteins were diluted at 0.32 mg/ml in 50 mM Hepes/KOH pH 8.0, 50 mM NaCl, 5 mM MgCl_2_, 0.035% DDM, and 0.03% sodium cholate. Where specified, samples were supplemented with 4 mM ATP and 0.2 mM sodium orthovanadate (Vi) and incubated à 30 °C for 20 min. Oxidation was started by addition of 0.2 mM copper(II) sulfate and 0.6 mM 1,10-phenanthroline. At 0, 1, 2, 5, 10, 15, 30, 45 and 60 min time points, 10 μl samples (3 μg BmrA) were then withdrawn from the reaction. For each sample NEM at a final concentration of 10 mM was then immediately added to stop the reaction. Proteins were analyzed by SDS-PAGE in non-reducing conditions.

#### Nanodiscs

BmrA was first reconstituted in nanodiscs as described (79), and samples at 0.4 mg/ml were reduced with 3.5 mM DTT for 10 min at 25 °C before being diluted at 0.08 mg/ml in 50 mM Hepes/KOH pH 8.0, 50 mM NaCl, and 5 mM MgCl_2_. Where specified samples were then supplemented with 4 mM ATP, and 0.2 mM sodium orthovanadate (Vi), and incubated à 30 °C for 20 min. Oxidation was started by addition of 0.2 mM copper(II) sulfate and 0.6 mM 1,10-phenanthroline. At 0, 1, 2, 5, 10, 15, 30, 45 and 60 min time points, samples of 20 μl (1.6 μg BmrA) were withdrawn from the reaction. For each sample, NEM at a final concentration of 10 mM was then immediately added to stop the reaction. Proteins were analyzed by SDS-PAGE in nonreducing conditions.

### Grid preparation for cryo-EM

A volume of 4 μl of detergent-purified WT BmrA or C436S/A582C mutant at 6.2 and 6.4 mg/ml, respectively, was applied onto a Quantifoil 300-mesh R1.2/1.3 grid that has been glow discharged in a PELCO easiGlow system for 30 s with a 15 mA current. Vitrification was performed in liquid ethane using a FEI Vitrobot Mark IV (Thermo Fischer Scientific) at 22 °C and 95% humidity with a blot force of −1 and blot time of 4 s.

### Single particle analysis acquisition and reconstruction

After clipping, the grid was imaged on a Titan Krios G4 TEM (Thermo Fisher Scientific) equipped with a K3 direct electron detector and energy filter (Gatan). The samples were imaged at a nominal magnification of 81,000x corresponding to a detector pixel size of 1.07 Å and defocus values ranging from −0.6 μm to −2 μm. A total of 13,937 and 3979 exposures of 50 e^-^/Å^2^ over 40 frames were collected for BmrA mutant and WT, respectively. Reconstruction was carried out using cryoSPARC v3.3.1 and v4.1.1 (Figs. S2 and S4) (80) (https://cryosparc.com).

For the C436S/A582C mutant dataset, 4,226,435 particles were extracted using automated blob picking following motion correction and contrast transfer function (CTF) estimation. Two rounds of 2D classification were performed and resulted in a working dataset of 2,057,656 particles. These set of particles were subjected to *ab initio* reconstruction to create a *de novo* model further refined using homogeneous 3D refinement with C1 symmetry. Refinement was then performed with a C2 symmetry, global CTF refinement, and local refinement with C2 symmetry applied. The canonical 3D refinement resulted in a 3.01 Å resolution map with a B-factor of 90.1. Resolution was estimated using the 0.143 gold standard Fourier shell correlation threshold, calculated using a relaxed solvent map. This subset of particles was then subject to 3D classification (10 classes) in C1. Classes reaching below 5 Å were subject to homogeneous refinement with C2 symmetry then to local refinement with C2 symmetry. The protein model (PDB 8CHB) was built on the final map which displayed the highest resolution at 3.14 Å with a B-factor of 77.3 (Electron Microscopy Data Bank EMDB-16659). The atomic model was first built with ModelAngelo (81), manually adjusted using Coot v0.9 (82) (https://www2.mrc-lmb.cam.ac.uk/personal/pemsley/coot/) and ISOLDE v1.4 (83) (https://isolde.cimr.cam.ac.uk/), and refined with PHENIX (84) (https://phenix-online.org/).

For the WT BmrA dataset, after motion correction and CTF estimation, a total of 3,972,311 particles were extracted using automated blob picking. A total of 1,043,614 particles were conserved after two rounds of 2D classification. This set of particles was used to build an *ab initio* model further refined using homogeneous 3D refinement in C2. The overall resolution of the final map was 3.16 Å with a B-factor of −111.6. To match the number of particles per class used for BmrA C436S/A582C mutant, the million particles were then divided into five classes by 3D classification in C1. The three classes showing less than 5 Å were then subjected to homogeneous refinement with C2 symmetry, then to local refinement in C2. Comparing the class with the best resolution of 3.16 Å with the initial map built with one million particles, differences in density were minimal. No extra structural feature was present and density seems to be slightly more defined in the NBDs for the initial map, which was then used to build the protein model. The protein structure (PDB 8QOE) was built using the mutant structure (PDB 8CHB), refined with Phenix, and manually adjusted with Coot and ISOLDE. For both protein structures validation was performed with MolProbity (85, 86). Data collection and refinement statistics are listed in Table S1.

### Site-directed spin labeling

Detergent-purified proteins (WT, C436S/T123C or C436S/A582C) were first concentrated and reduced in the presence of 100 M excess of DTT in a glove box. After 30 min incubation, the DTT was removed using a desalting PD-10 column (GE HealthCare) and the elution buffer D_2_O 50 mM Hepes/NaOH pH 8.0, 50 mM NaCl, 0.035% DDM, 0.03% sodium cholate, and 10% d_8_-glycerol. Next, labeling was performed in a glove box with the 1-oxyl-2,2,5,5-tetramethylpyrroline-3-methyl methanethiosulfonate spin label (MTSL) according to optimized procedures (87, 88). In short, 10 M excess of MTSL from a stock solution at 43 mM in acetonitrile was added to the protein sample. After 1 h incubation the excess of free MTSL was removed using a second desalting column and the same elution buffer. Fractions containing the labeled protein were identified by continuous wave (cw) EPR at room temperature, pooled and concentrated using 100 kDa molecular weight cut-off Vivaspin 2 (Sartorius) to a monomer concentration of 80 to 100 μM. Labeling yields were estimated as described previously (87, 88) and were found to be in the range of 90 to 100% per monomer.

### EPR measurements

The labeled proteins were prepared to a final concentration of 100 μM in the same deuterated buffer that was used for spin labeling with an additional 10% v/v d_8_-glycerol. For the preparation of vanadate-trapped samples, a 10-fold molar excess of orthovanadate was added, and the sample homogenized before addition of 100-fold molar excess of Mg^2+^ and ATP. The sample was then incubated for 15 min at room temperature and placed in a Q-band EPR quartz tube. Q-band (35 GHz) pulsed EPR experiments were performed on an Elexsys E580 spectrometer (Bruker) using an EN 5107D2 resonator and equipped with a helium cryostat CF935 (Oxford instruments). Temperature was reached with a closed cycle Helium Stinger system (Bruker) and controlled by a Mercury controller (Oxford instruments). The data were recorded at a temperature of 60 K. A four-pulse DEER sequence (89) was used with pulse durations of 20 ns (p/2 pulse) and 40 ns (p pulse) with delays t1 of 200 ns and t2 adjusted according to Tm (phase memory time). The pump frequency was set to the maximum of the resonance in the echo field sweep spectrum and the observed frequency was 56 MHz higher. To suppress undesirable echoes an eight-step phase cycle was applied. Data were collected for about 12 to 18 h for each sample. Data from DEER traces were extracted by using the DeerAnalysis 2019 software and processed with Tikhonov regularization to obtain the interspin distributions (90) (https://epr.ethz.ch/software/older-versions/old_deeranalysis.html). Homogenous 3D-background function and neural network correction (DEERnet) were chosen for the treatment of experimental dipolar evolution datasets. The regularization factor α was chosen according to the L-curve criterion, based on a compromise between smoothness and resolution (91).

## Data availability

All relevant data are within the paper and its supporting information. DEER data have been deposited on entrepot.recherche.data.gouv.fr (https://doi.org/10.57745/AVNAUP). The atomic models and the electron microscopy maps have been deposited in the Protein Data Bank under the accession codes PDB 8QOE and Electron Microscopy Data Bank EMDB-18535 for WT BmrA and PDB 8CHB/EMBD-16659 for the C436S/A582C cross-linked mutant.

## Supporting information

This article contains supporting information (90).

## Conflict of interest

The authors declare that they have no conflicts of interest with the contents of this article.

## Supporting information

Movie S4

Supplemetal information
