# Supplementary material for: The transport activity of the multidrug ABC transporter BmrA does not require a wide separation of the nucleotide-binding domains
Source: J Biol Chem. 2023 Dec 9;300(1):105546. doi: 10.1016/j.jbc.2023.105546 (PMC10821409; doi:10.1016/j.jbc.2023.105546)
Supplement: Supplemetal information [file mmc5.docx]

**The transport activity of the multidrug ABC transporter BmrA does not require a wide separation of the nucleotide-binding domains**

Margot Di Cesare ^1^, Elise Kaplan ^1^, Julia Rendon ^2^, Guillaume Gerbaud ^2^, Sepideh Valimehr^3^, Alexia Gobet ^4^, Thu-Anh Thi Ngo ^1^, Vincent Chaptal ^4^, Pierre Falson ^4^, Marlène Martinho ^2^, Pierre Dorlet ^2^, Eric Hanssen ^3^, Jean-Michel Jault ^1,^* and Cédric Orelle ^1,^*

^1^ Bacterial Nucleotide-Binding Proteins Team, Molecular Microbiology and Structural Biochemistry (MMSB), UMR 5086 CNRS/University of Lyon, Lyon, France

^2^ CNRS, Aix-Marseille Université, BIP, IMM, Marseille, France

^3^ Ian Holmes Imaging Center and Department of Biochemistry and Pharmacology and ARC centre for Cryo-Electron Microscopy of Membrane Proteins, Bio21 Institute, University of Melbourne, Parkville, VIC 3010, Australia

^4^ Drug Resistance and Membrane Proteins Team, Molecular Microbiology and Structural Biochemistry (MMSB), UMR 5086 CNRS/University of Lyon, Lyon, France

***** Correspondence: jean-michel.jault@ibcp.fr; cedric.orelle@ibcp.fr

**Supplementary information**

**This PDF file includes:**

Table S1

Figures S1 to S9

Legends for Movies S1 to S4

SI Reference

**Other supplementary materials for this manuscript include the following:**

Movies S1 to S4

| **Table S1. Cryo-EM statistics** |  |  |
| --- | --- | --- |
|  | **BmrA wild-type** | **BmrA C436S/A582C** |
|  | **PDB 8QOE / EMDB-18535** | **PDB 8CHB / EMDB-16659** |
| **Data collection** |  | |
| Microscope | FEI Titan Krios | FEI Titan Krios |
| Voltage (kV) | 300 | 300 |
| Camera | Gatan K3 infinium | Gatan K3 infinium |
| Mode | Counting | Counting |
| Magnification | 81,000 | 81,000 |
| Pixel size (Å) | 1.07 | 1.07 |
| Electron dose (e–/Å^2^) | 50 | 50 |
| Defocus range (μm) | -0.6 to -2.0 | -0.6 to -2.0 |
| Exposure (s) | 3.8 | 3.8 |
|  |  |  |
| **Reconstruction** |  |  |
| Software | CryoSPARC-3.3.1 & 4.1.1 | CryoSPARC-3.3.1 & 4.1.1 |
| Frames | 40 | 40 |
| Symmetry | C2 | C2 |
| Initial particle images (no.) | 3,972,311 | 4,226,435 |
| Final particle images (no.) | 1,043,614 | 2,057,656 |
| Map resolution, FSC_0.143_ (Å) | 3.16 | 3.14 |
| Map-sharpening B factor (Å) | -111.6 | -77.3 |
|  |  |  |
| **Refinement** |  | |
| Software | Phenix-1.20.1 | Phenix-1.20.1 |
| Map resolution, FSC_0.5_ (Å) | 3.2 | 3.4 |
|  |  |  |
| **Model composition** |  |  |
| Non-hydrogen atoms | 8,744 | 8,916 |
| Protein residues | 1,136 | 1,158 |
|  |  |  |
| **R.m.s. deviations** |  |  |
| Bond lengths (Å) | 0.018 | 0.012 |
| Bond angles (°) | 2.486 | 1.847 |
|  |  |  |
| **Validation** |  |  |
| MolProbity score | 1.43 | 1.57 |
| Clashscore | 4.38 | 3.80 |
| Poor rotamers (%) | 0.84 | 1.64 |
|  |  |  |
| **Ramachandran plot** |  |  |
| Favored (%) | 96.64 | 96.36 |
| Allowed (%) | 3.36 | 3.64 |
| Disallowed (%) | 0.00 | 0.00 |


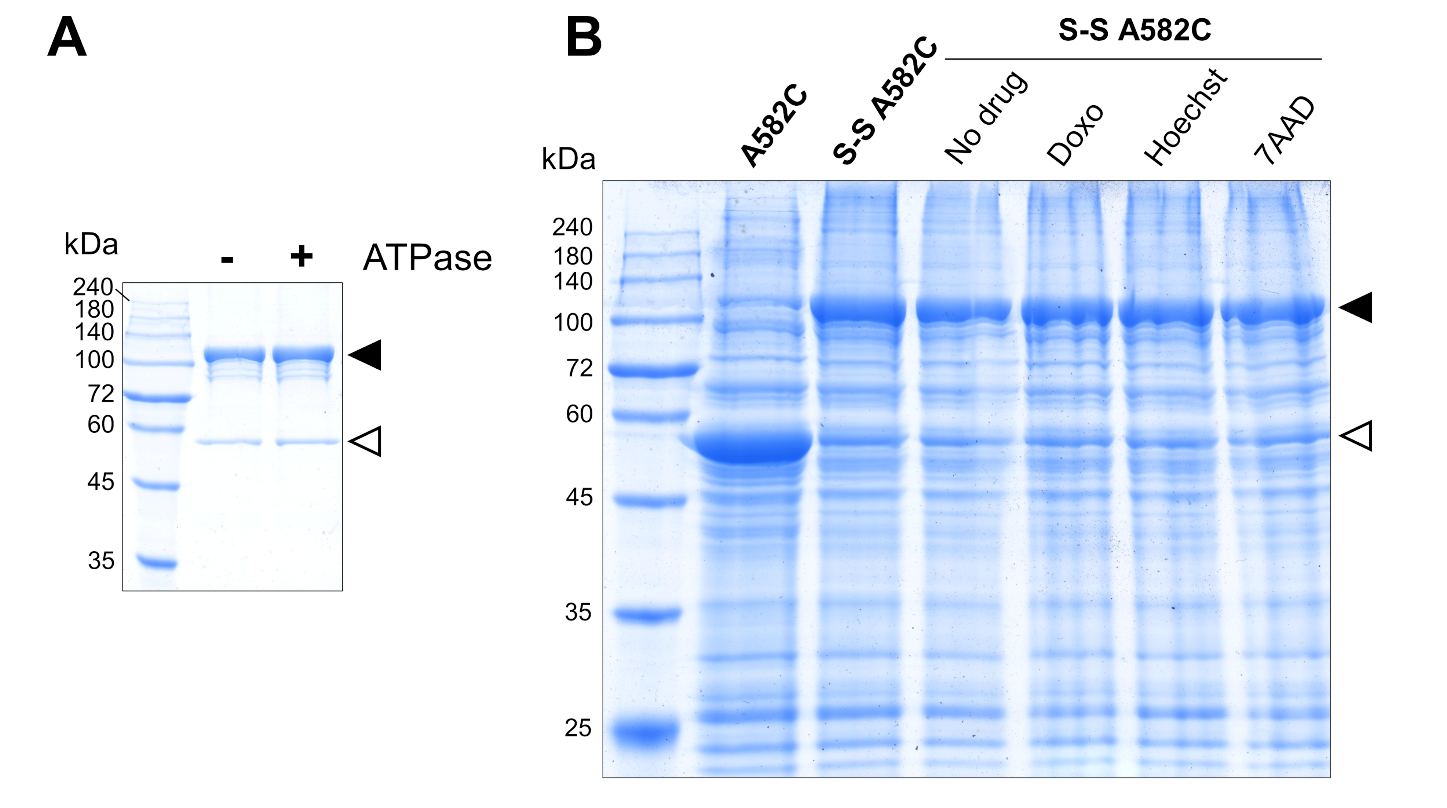


**Figure S1. Presence of the disulfide bond in A582C cross-linked mutant after ATPase activity or drug transport. (A)** SDS-PAGE analysis of detergent-purified A582C mutant (3 µg were loaded) treated with an oxidative agent (- ATPase) and further incubated in an ATPase assay medium during 10 min (+ ATPase). The arrowheads show the position of the BmrA monomer (white color) and the S-S cross-linked BmrA dimer (black color). **(B)** SDS-PAGE analysis of inverted membrane vesicles (20 µg were loaded) containing non-treated A582C mutant (A582C), treated with an oxidative agent (S-S A582C), or treated with an oxidative agent and used for drug transport assay (S-S A582C + drugs) with the indicated drug (doxo, doxorubicin; Hoechst, Hoechst 33342 and 7AAD, 7-amino-actinomycin D). A control was also incubated in the solution used for the transport assay without any drug (no drug).


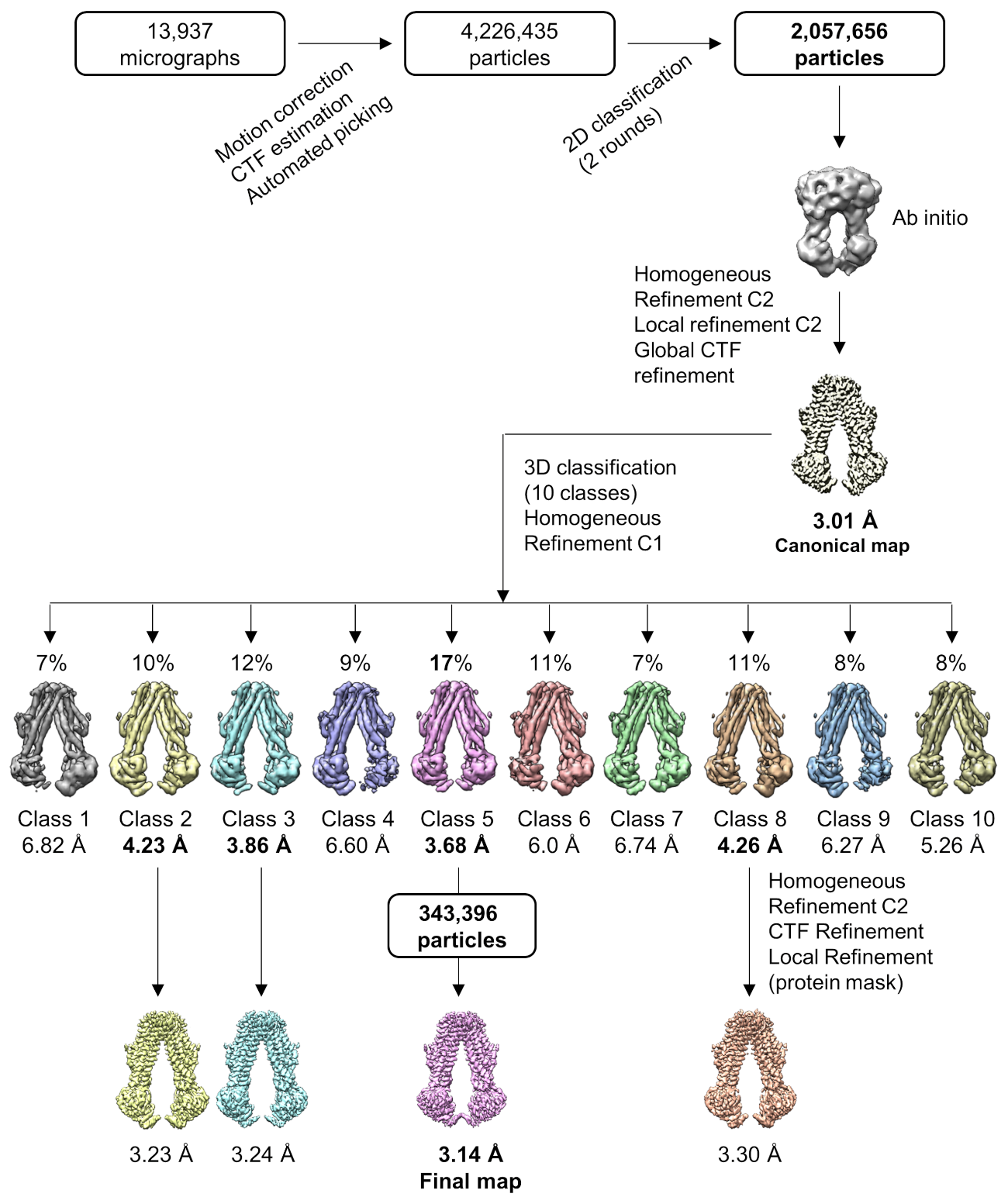


**Figure S2. Cryo-EM processing workflow for C436S/A582C cross-linked BmrA mutant.** Single-particle cryo-EM data were processed using cryoSPARC. After motion correction, CTF estimation, and automated blob picking, two rounds of 2D classification were performed to remove bad particles. An *ab initio* model was built and used to generate a *de novo* model further refined in C2 using homogeneous 3D refinement, global CTF refinement, and local refinement with the 2 million remaining particles. The canonical 3D refinement resulted in a 3.01 Å resolution map, yet less information was present in the C-terminal region. Therefore, this subset of particles was then subject to 3D classification (10 classes) in C1. Classes 2, 3, 5 and 8 reaching below 5 Å were subject to C2 refinement and local refinement. Density covering the disulfide bridge at position 582 was present for the four maps. The protein model (PDB 8CHB) was built on the map at 3.14 Å generated from class 5 (EMDB-16659) which displays the highest resolution.


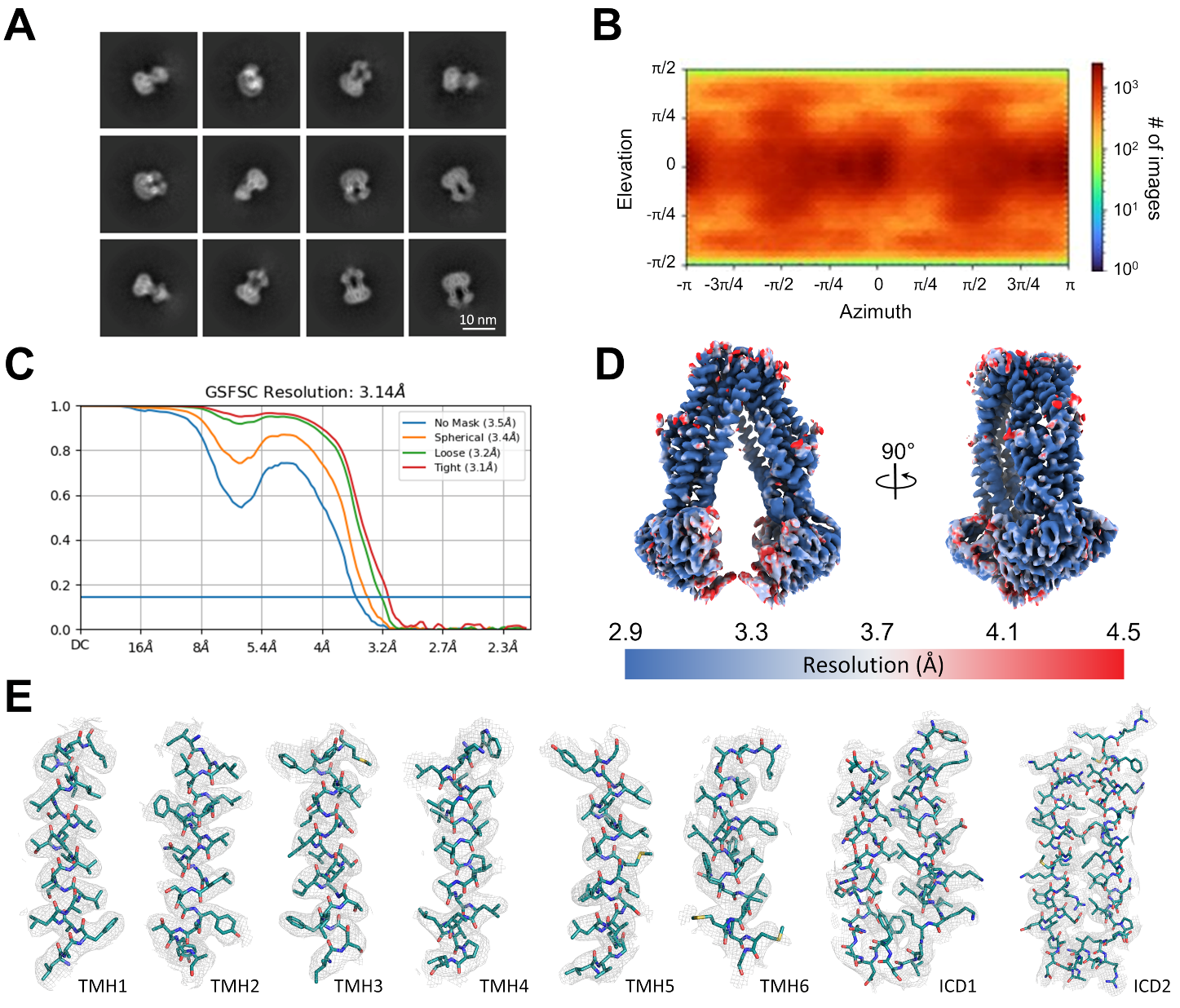


**Figure S3. Cryo-EM analysis of C436S/A582C cross-linked BmrA mutant. (A)** 2D class averages. **(B)** Angular distribution plot of particles. **(C)** Orientation Gold-standard Fourier Shell Correlation (FSC) curves for the final 3D reconstruction. **(D)** EM volume map colored according to the local resolution. **(E)** Representative density for transmembrane helices (TMH) and intracellular domains (ICD).


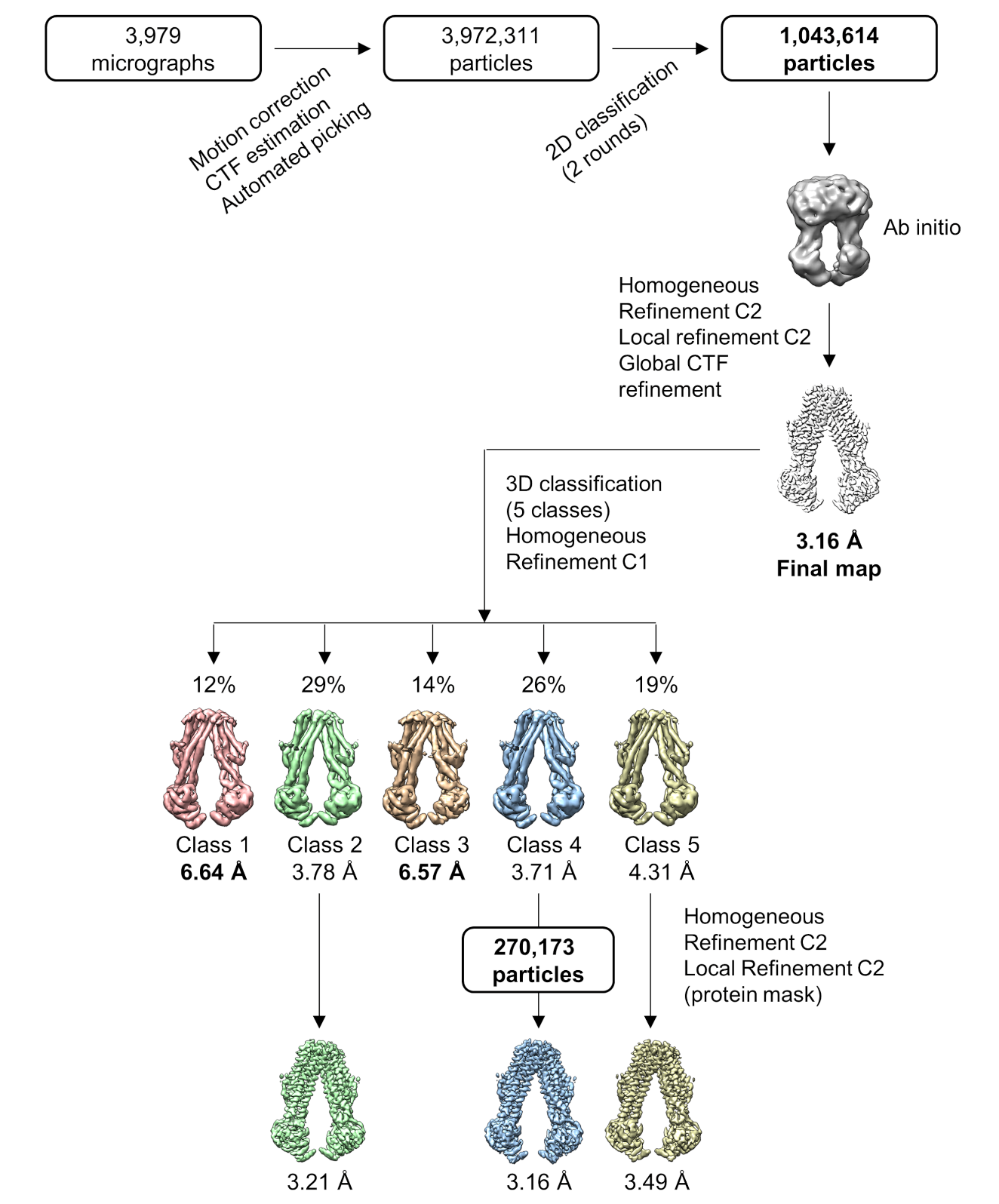


**Figure S4. Cryo-EM processing workflow for wild-type BmrA.** Single-particle cryo-EM data were processed using cryoSPARC. After motion correction, CTF estimation, and automated blob picking, two rounds of 2D classification were performed to remove bad particles. An *ab initio* model was built and used to generate a *de novo* model further refined in C2 using homogeneous 3D refinement, global CTF refinement and local refinement with the one million remaining particles, leading to a 3.16 Å resolution map. As performed for BmrA mutant, the particles were then subject to 3D classification (5 classes) in C1. Classes 2, 4 and 5 reaching below 5 Å were subject to C2 refinement and local refinement. The best map presented identical resolution as the initial map but with a slightly less defined density in the NBDs and no extra structural feature present. The initial map, built from a larger number of particles, was then used to build the protein model (PDB 8QOE) and for deposition (EMDB-18535).


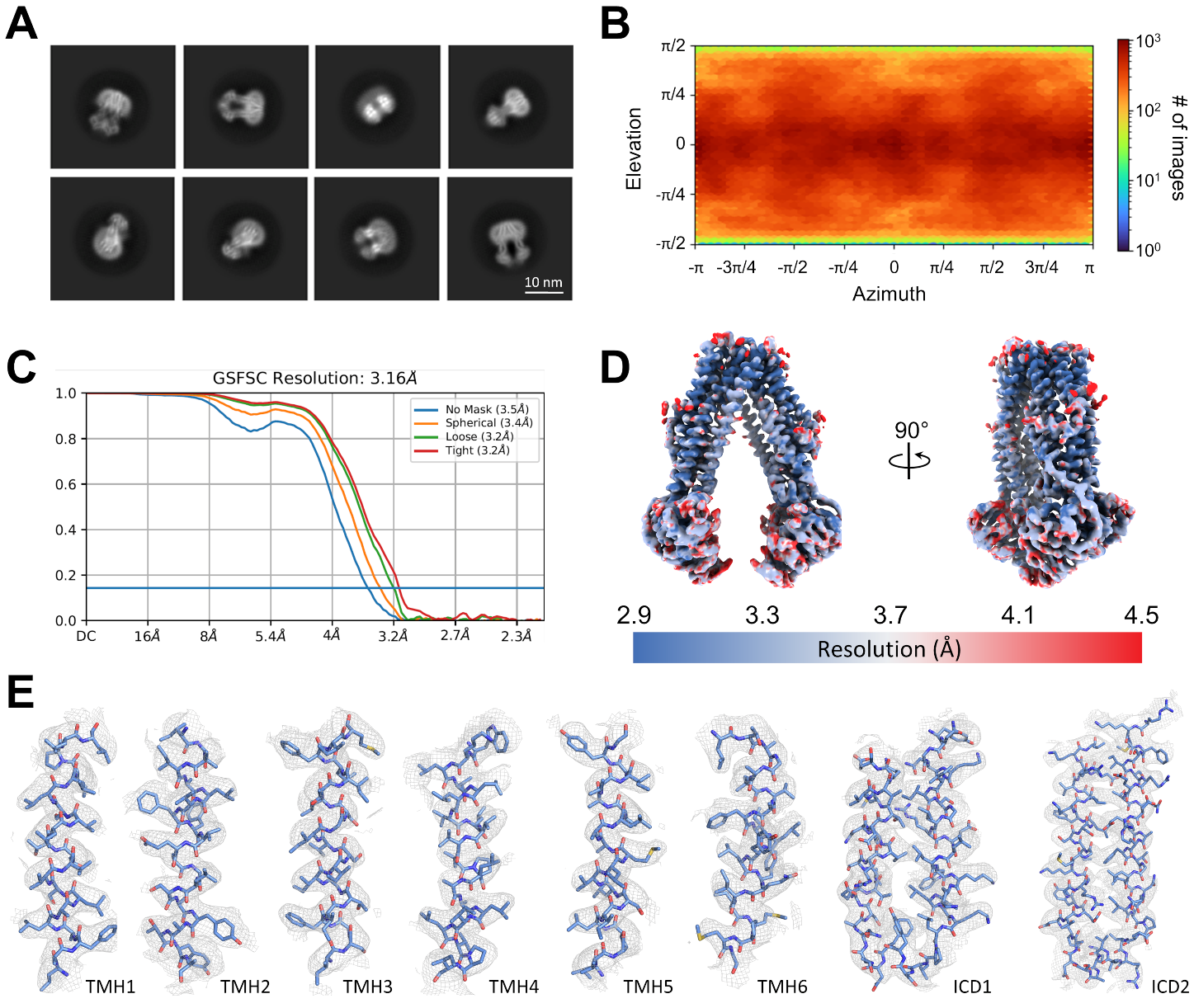


**Figure S5. Cryo-EM analysis of wild-type BmrA. (A)** 2D class averages. **(B)** Angular distribution plot of particles. **(C)** Orientation Gold-standard Fourier Shell Correlation (FSC) curves for the final 3D reconstruction. **(D)** EM volume map colored according to the local resolution. **(E)** Representative density for transmembrane helices (TMH) and intracellular domains (ICD).


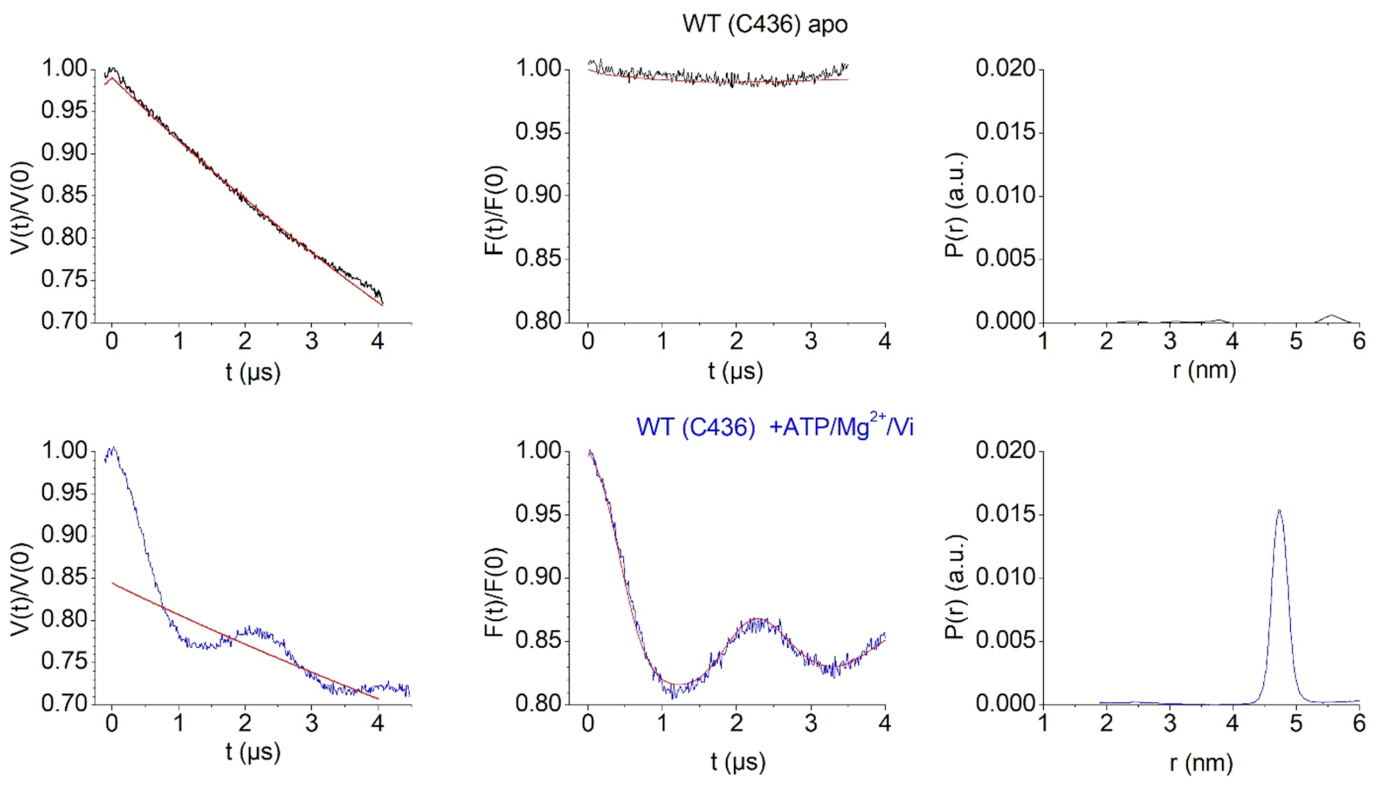


**Figure S6. Experimental Q-band DEER traces recorded at 60K for BmrA wild-type labeled at position 436**. Black traces (top panels) are for the apo protein (IF conformation) and blue traces (bottom panels) are for the protein trapped by addition of ATP-vanadate (OF conformation). On the left panels the red lines indicate the baselines used for background correction. The resulting corrected DEER traces are shown in the middle panels with superimposed fits (in red) derived from Tikhonov regularization using DeerAnalysis 2019 (1). The Tikhonov-derived distance distributions are shown on the right panels.


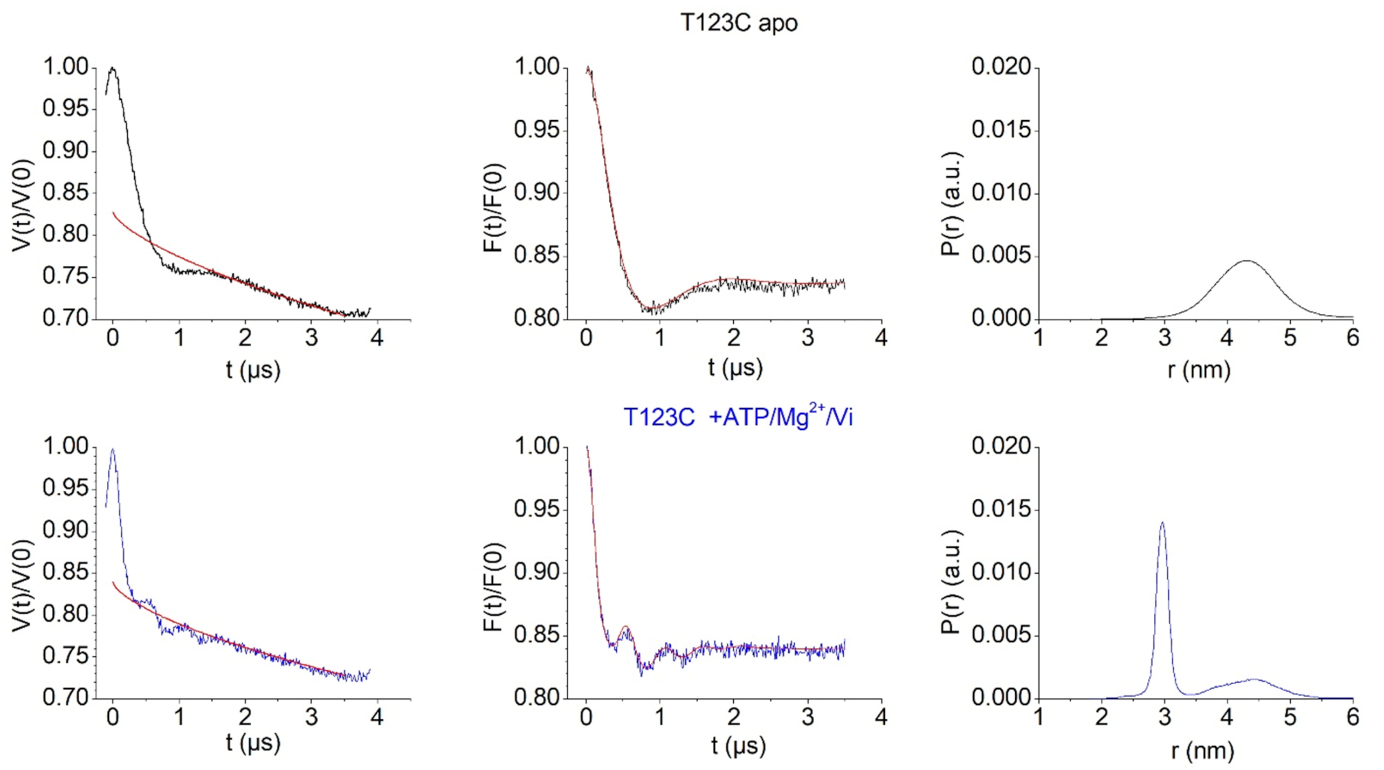


**Figure S7. Experimental Q-band DEER traces recorded at 60K for BmrA C436S/T123C mutant labeled at position 123**. Black traces (top panels) are for the apo protein (IF conformation) and blue traces (bottom panels) are for the protein trapped by addition of ATP-vanadate (OF conformation). On the left panels the red lines indicate the baselines used for background correction. The resulting corrected DEER traces are shown in the middle panels with superimposed fits (in red) derived from Tikhonov regularization using DeerAnalysis 2019 (1). The Tikhonov-derived distance distributions are shown on the right panels.


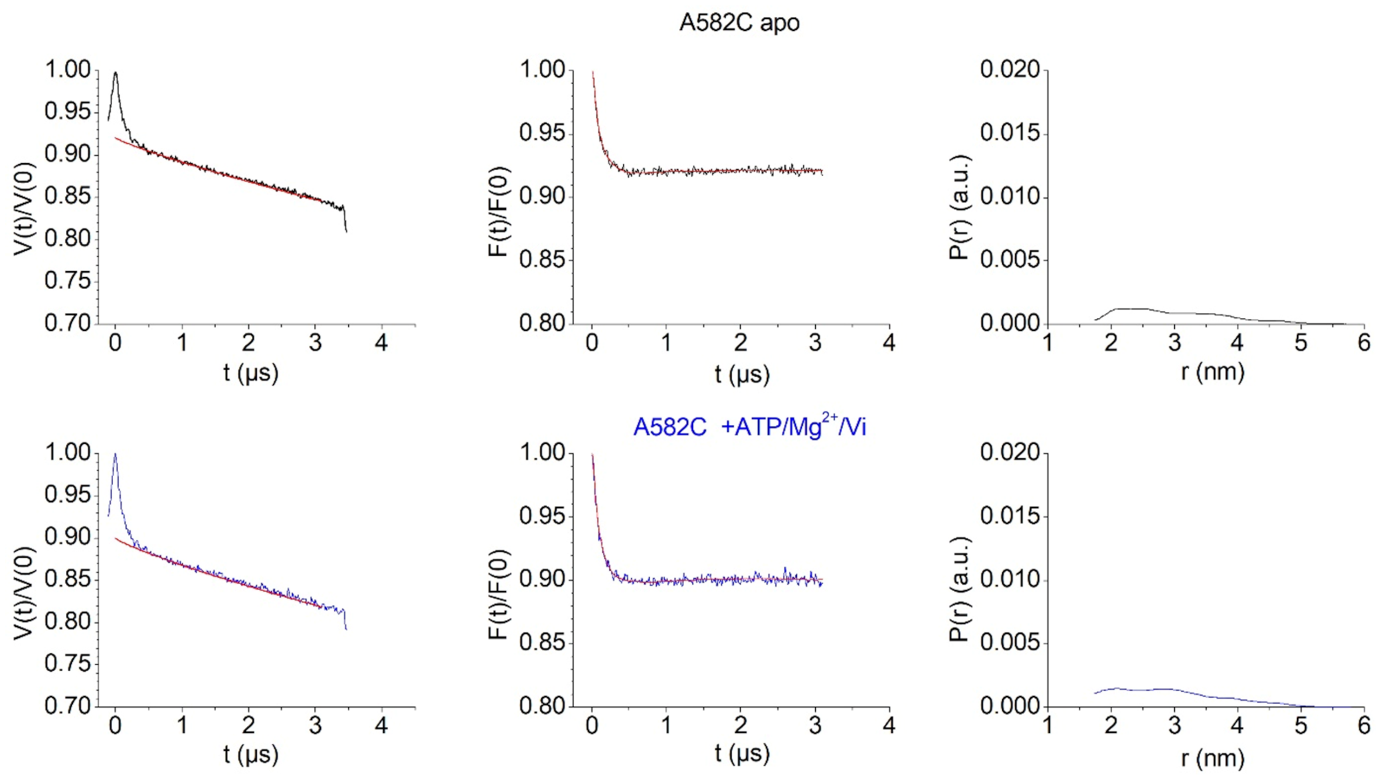


**Figure S8. Experimental Q-band DEER traces recorded at 60K for BmrA C436S/A582C mutant labeled at position 582**. Black traces (top panels) are for the apo protein (IF conformation) and blue traces (bottom panels) are for the protein trapped by addition of ATP-vanadate (OF conformation). On the left panels the red lines indicate the baseline used for background correction. The resulting corrected DEER traces are shown in the middle panels with superimposed fits (in red) derived from Tikhonov regularization using DeerAnalysis 2019 (1). The Tikhonov-derived distance distributions are shown on the right panels.


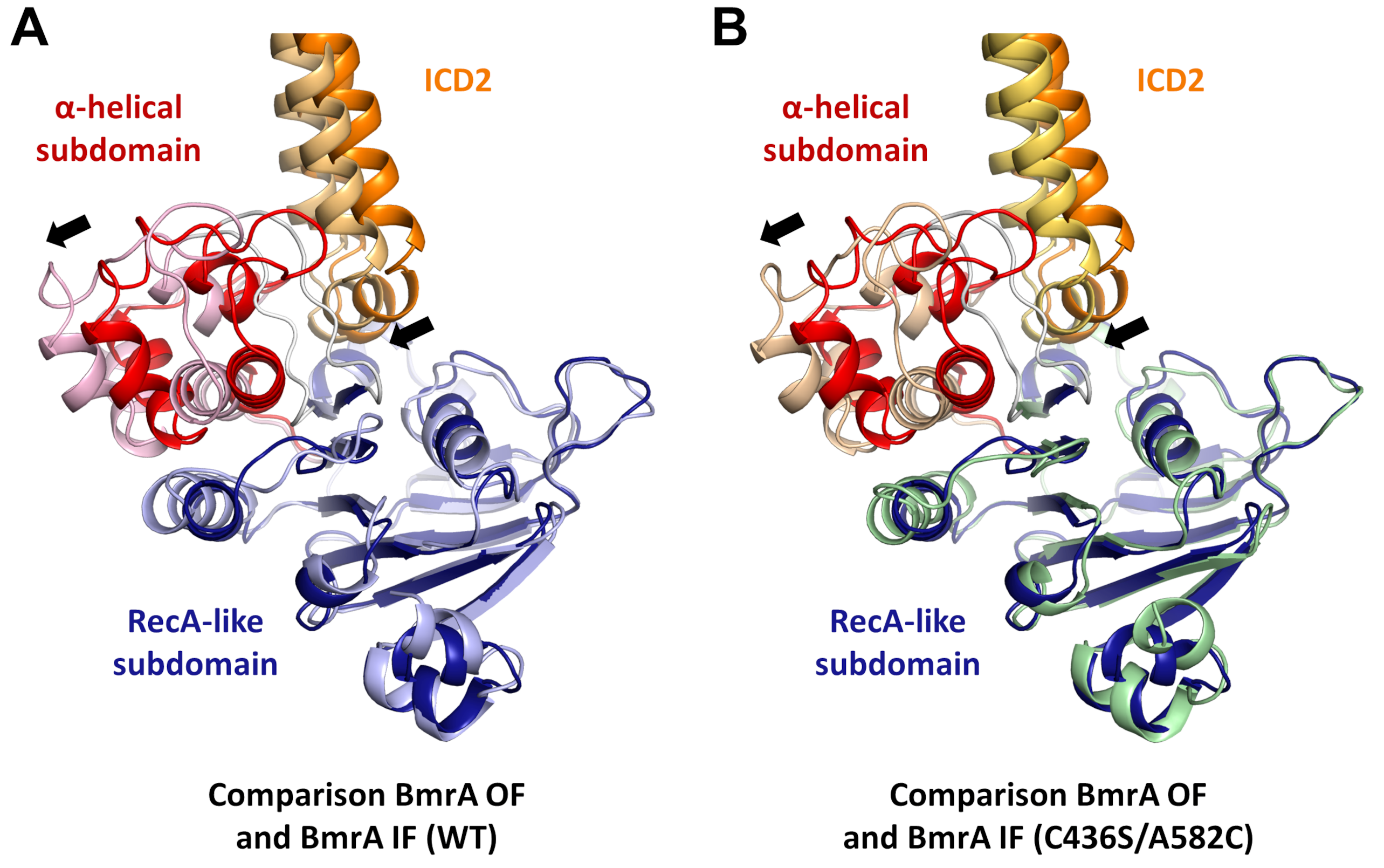


**Figure S9. Conformational changes at the NBD/TMD interface of BmrA.** Structures of OF (dark hues, 7OW8) and IF conformations of BmrA wild-type (**A**, 8CHB) or C436S/A582C cross-linked mutant (**B**, 8QOE) were aligned based on the RecA-like subdomain (RecA, blue) of the NBD. During the transition between the IF and OF states, a rotation of the helical subdomain (helical, red) relative to the RecA-like subdomain of the NBD induces a reorientation of the coupling helix (ICD2, yellow-orange).

**Supplementary movies**

**Movie S1. Structural alignment of BmrA wild-type and C436S/A582C mutant.** Inward-facing conformation of BmrA wild-type (blue) and BmrA C436S/A582C (green). The first part of the movie shows a 360° rotation along the *y*-axis. In the second part a close-up zoom is realized on several NBD residues (Q483, H535, K380 and Y350).

**Movie S2. Flexibility of BmrA transporter.** Morph of different maps generated in cryoSPARC refinement for BmrA C436S/A582C mutant (left, green) and wild-type (right, blue). The movie shows the transition from the most closed to the most open map.

**Movie S3. Molecular morph highlighting the NBD tweezers-like closure motion in BmrA and comparison with MsbA (profile and bottom views).** Left: morph of BmrA transiting from the inward-facing (C436S/A582C mutant, 8CHB) to the outward-facing conformation (E504A mutant, 7OW8). Right: same animation with the MsbA transporter using the structures corresponding to the inward- and outward-facing states (6BL6 and 3B5Z, respectively). For each protein a bottom-up view of the transporter is shown at the top and a profile view at the bottom. Secondary elements of the nucleotide-binding sites are highlighted and colored as specified.

**Movie S4. Molecular morph highlighting the NBD tweezers-like closure motion in BmrA and comparison with MsbA (side and top views).** Left: morph of BmrA transiting from the inward-facing (C436S/A582C mutant, 8CHB) to the outward-facing conformation (E504A mutant, 7OW8). Right: same animation with the MsbA transporter using the structures corresponding to the inward- and outward-facing states (6BL6 and 3B5Z, respectively). For each protein a bottom-up view of the transporter is shown at the top and a profile view at the bottom.

**Reference**

1. Jeschke, G., Chechik, V., Ionita, P., Godt, A., Zimmermann, H., Banham, J., Timmel, C. R., Hilger, D., and Jung, H. (2006) DeerAnalysis2006—a comprehensive software package for analyzing pulsed ELDOR data. *Applied Magnetic Resonance* **30**, 473-498
